# Real-world gait speed estimation, frailty and handgrip strength: a cohort-based study

**DOI:** 10.1038/s41598-021-98359-0

**Published:** 2021-09-23

**Authors:** Abolfazl Soltani, Nazanin Abolhassani, Pedro Marques-Vidal, Kamiar Aminian, Peter Vollenweider, Anisoara Paraschiv-Ionescu

**Affiliations:** 1 Laboratory of Movement Analysis and Measurement (LMAM) , Ecole Polytechnique Federale de Lausanne (EPFL), Lausanne, Switzerland; 2 Department of Medicine, Internal Medicine, Lausanne University Hospital (CHUV) and University of Lausanne (UNIL), Lausanne, Switzerland

**Keywords:** Health care, Medical research, Risk factors, Engineering

## Abstract

Gait speed is a reliable outcome measure across multiple diagnoses, recognized as the 6th vital sign. The focus of the present study was on assessment of gait speed in long-term real-life settings with the aim to: (1) demonstrate feasibility in large cohort studies, using data recorded with a wrist-worn accelerometer device; (2) investigate whether the walking speed assessed in the real-world is consistent with expected trends, and associated with clinical scores such as frailty/handgrip strength. This cross-sectional study included *n* = 2809 participants (1508 women, 1301 men, [45–75] years old), monitored with a wrist-worn device for 13 consecutive days. Validated algorithms were used to detect the gait bouts and estimate speed. A set of metrics were derived from the statistical distribution of speed of gait bouts categorized by duration (short, medium, long). The estimated usual gait speed (1–1.6 m/s) appears consistent with normative values and expected trends with age, gender, BMI and physical activity levels. Speed metrics significantly improved detection of frailty: AUC increase from 0.763 (no speed metrics) to 0.798, 0.800 and 0.793 for the 95th percentile of individual’s gait speed for bout durations < 30, 30–120 and > 120 s, respectively (all p < 0.001). Similarly, speed metrics also improved the prediction of handgrip strength: AUC increase from 0.669 (no speed metrics) to 0.696, 0.696 and 0.691 for the 95th percentile of individual’s gait speed for bout durations < 30, 30–120 and > 120 s, respectively (all p < 0.001). Forward stepwise regression showed that the 95th percentile speed of gait bouts with medium duration (30–120 s) to be the best predictor for both conditions. The study provides evidence that real-world gait speed can be estimated using a wrist-worn wearable system, and can be used as reliable indicator of age-related functional decline.

## Introduction

Gait speed, recognized as the ‘6th vital sign’^1,2^, is a reliable and sensitive measure of people’s functional ability, closely associated with well-being, healthy aging, physical frailty and survival in older adult populations^3–14^. Most studies that have contributed to the accumulated evidence about reliability of gait speed as predictor of adverse health outcomes among community-dwelling adults were based on measurements in the laboratory/clinical settings. However, mounting evidence indicates that the speed assessed in a laboratory setting does not fully reflect the speed of individuals in their everyday life context^15–20^. Therefore, there is growing consensus about necessity to develop and validate tools for assessment of gait in real-world conditions^15,18,19,21–24^.

Gait/locomotion is an essential dimension of daily physical activity (PA) behavior. Monitoring systems including body-worn sensor devices (e.g., accelerometers) and dedicated data processing algorithms may allow to characterize the multiple dimensions of PA, such as *type* (gait, body postures), *duration* of bouts, and *intensity* (*speed,* accelerometer counts*,* metabolic equivalent of task). One requirement for speed estimation in long-term monitoring protocols is detection of gait bouts. An additional constraint is to develop algorithms based on minimal data, usually from an unobtrusive single body worn device. The most common single device locations for long-term monitoring are the trunk (e.g., waist, chest, hip, lower back) or the wrist. While classification of PA dimensions using wrist movement data is technically challenging, recent research focused on the development and validation of robust algorithms^25–27^.

Given the difficulty of an accurate estimation of gait speed in real-world situations, most existing commercial wearable devices still deploy the more straightforward classical approach for PA assessment, by providing only an estimation of PA intensity levels (time spent in sedentary, light, moderate, and vigorous intensities). The issue with this approach is that the processing of raw data (acceleration signal) and definition of thresholds to classify intensity levels differ between various devices, making difficult comparison of PA-related outcome measures across studies/clinical populations^28,29^. The assessment of PA using gait speed as an outcome measure may overcome this issue since walking/gait characterized by speed is an 'invariant' component of PA. Yet, there are additional aspects that need to be considered when real-world gait speed is used as an outcome measure. For example, only a few studies have investigated the gait parameters for different bout durations (e.g., short, medium, long)^30,31^. No study has evaluated the metrics derived from the statistical distribution of speed (e.g., mean, median, mode, standard deviation, etc.) in different gait bout durations, which are characteristic of PA behavior in daily-life context. Yet, the identification of bout durations corresponding to various purposeful daily activities and expected to carry relevant information about an individual’s physical performance, is an essential topic for outcome evaluation in population-based studies^30^.

Another aspect of being considered is the pervasiveness of real-world gait speed monitoring. The Global Navigation Satellite System (GNSS) integrated in smartphone could accurately measure gait speed^32^, but it is available only outdoors, and so only few values might be available per day, increasing the intra/inter subjects’ variability. Finally, an additional limitation of most existing studies is the relatively modest sample size, which reduces the power of the statistical analyses and generalization of the findings in large populations.

This cohort study targeted two primary goals. First, to demonstrate the feasibility of assessing gait speed using an accurate, user-friendly wrist-worn system^26,27^ for a long duration (13 consecutive days, 24 h per day) in a large population sample in free-living conditions. Second, to investigate the association of estimated real-world gait speed with subject characteristics, and clinical variables recognized as indicators of overall physical health and mobility, such as frailty condition and handgrip strength. Indeed, grip strength is a component of frailty phenotype^33^ and is considered as an indispensable biomarker for older people^34^. Hence, an analysis of the associations between grip strength and walking metrics among elderly (> 65 years) people was also conducted.

## Results

### Study participants

Of the initial 4881 participants in CoLaus|PsyCoLaus cohort (https://www.colaus-psycolaus.ch/professionals/colaus/), 1982 (no walking speed data) and 90 (missing covariates) were excluded, and 2809 (46.3% men, 53.7% women, mean age 62.4 ± 9.9 years) were included in this study. After analysis, data of each participant was verified to check the number of days with detected gait bouts (valid days); days with no bouts detected were discarded. From the 2809 subjects, ~ 98% had at least eight valid days, thus including both weekdays and weekend day(s). This number was large enough for the estimation of preferred gait speed and related metrics^35,36^. The characteristics of the participants included and excluded are reported in Table 1.

**Table 1:** Characteristics of participants included in this study (CoLaus cohort, Lausanne, Switzerland, 2014–2017).

Factors	Included	Excluded	*p*-value
Total	2809	2072	
**Gender**			0.021
Men	1301 (46.3)	891 (43.0)	
Women	1508 (53.7)	1181 (57.0)	
**Age (years)**			< 0.001
[45–54]	830 (29.6)	516 (24.9)	
[55–64]	909 (32.4)	593 (28.6)	
[65–75]	729 (25.9)	570 (27.5)	
75+	341 (12.1)	393 (19.0)	
**Body mass index categories**			< 0.001
Normal + underweight	1157 (41.2)	700 (33.8)	
Overweight	1140 (40.6)	635 (30.6)	
Obese	512 (18.2)	337 (16.3)	
Missing data	0 (0)	400 (19.3)	
**Physically active**			< 0.001
Active	1091 (38.8)	66 (3.2)	
Inactive	1718 (61.2)	92 (4.5)	
Missing data	0 (0)	1914 (92.3)	
**Frailty**			< 0.001
No	2552 (90.8)	1376 (66.4)	
Yes	257 (9.2)	209 (10.1)	
Missing data	0 (0)	487 (23.5)	
Handgrip strength (kg)	34.4 ± 12.0	32.7 ± 12.2	< 0.001

Results are expressed as the number of participants (column percentage) or average ± standard deviation. Between-group comparisons performed using chi-square and student’s t-test.

### Real-world gait bouts detection and speed estimation

**Figure 1:**
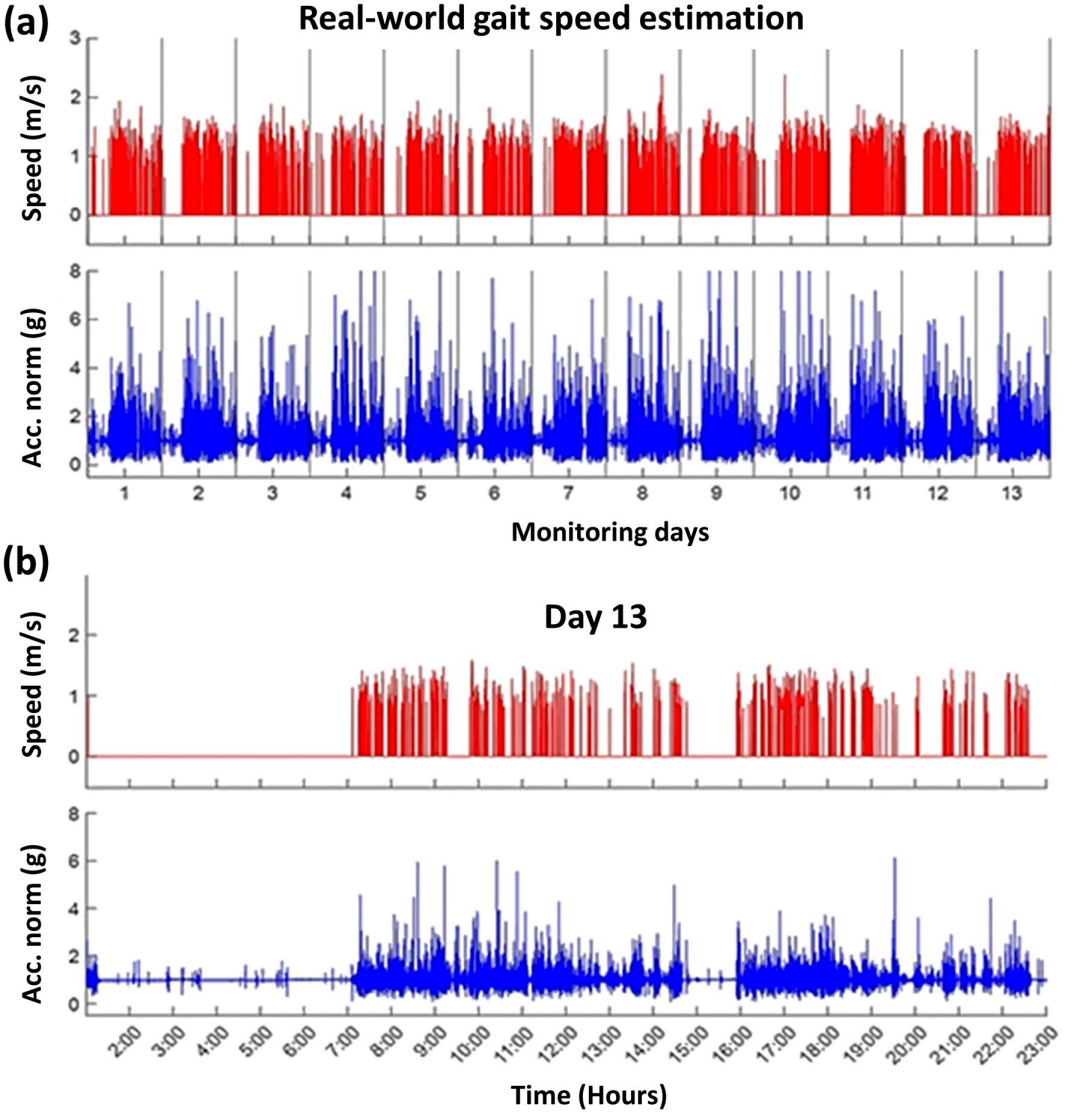
An illustrative example of a real-world GS estimation for a representative subject monitored in daily life situations. **(a)** Estimated speed of detected GB (red) and wrist acceleration norm (blue) during 13 continuous days; **(b)** one typical day (day #12), (CoLaus cohort, Lausanne, Switzerland, 2014–2017).

An illustrative example of wrist acceleration-based gait speed estimation for one study participant is shown in Fig. 1. Panel (a) displays the acceleration norm, and the estimated speed for the gait bouts detected during 13 continuous days in free-living situations. Panel (b) is a zoom to show analysis results in a typical day (24 h). This example demonstrates that the algorithms successfully estimated a consistent sequence of bouts and their corresponding speed in regular daily life activity. A repetitive pattern of gait bouts among successive days can be observed. Each day starts with a non-active period, likely related to sleep time, followed by two active periods in the morning and the afternoon (probably associated with a daily routine). Sometimes, a few bouts can be observed in the evening.

### Univariate analysis: real-world gait speed and subject characteristics

**Figure 2:**
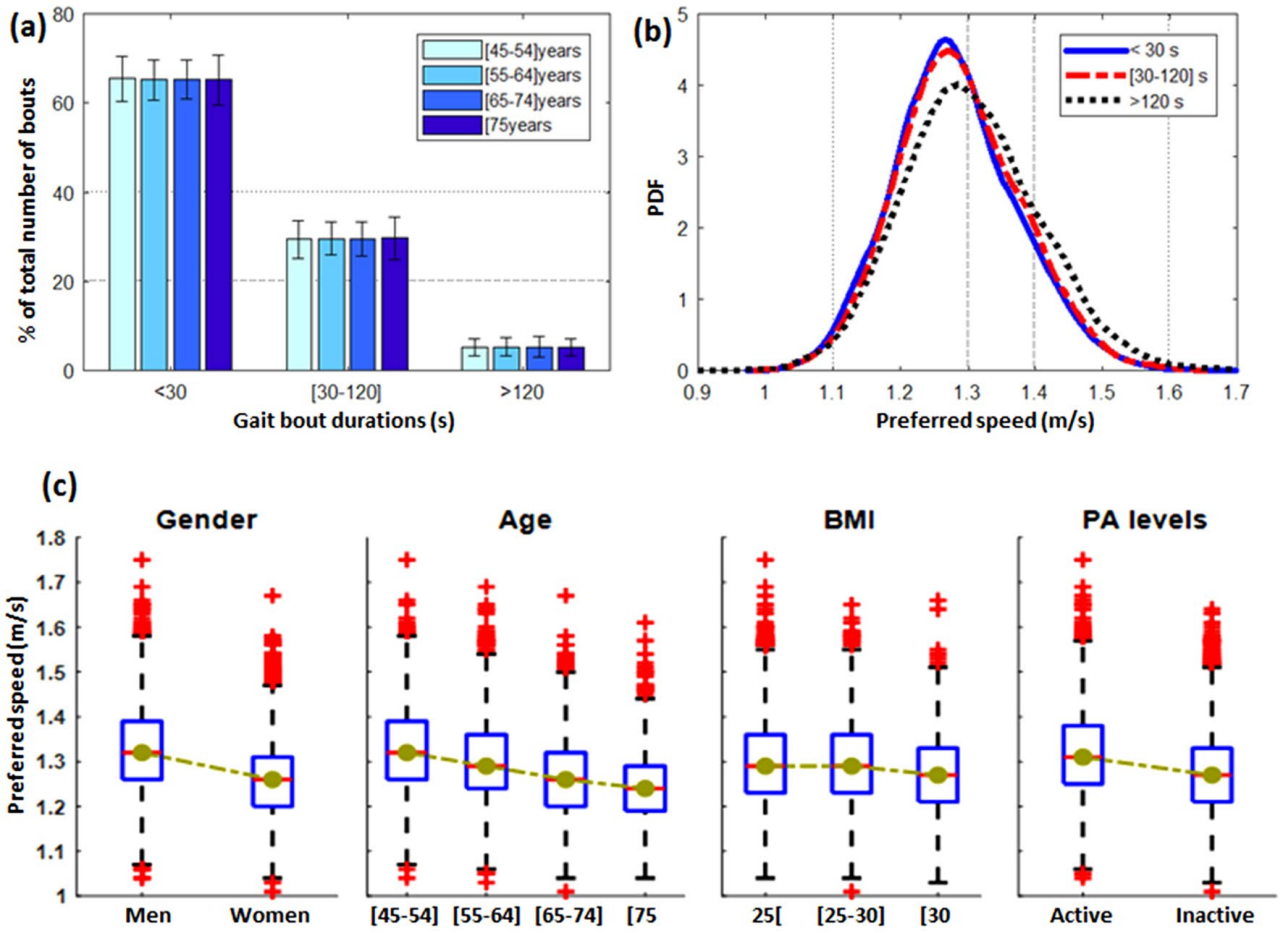
**(a)** Distribution of bouts within each duration category, stratified by age groups. Each bar and the corresponding error bar report the mean and SD. For each individual in each age group, the values were calculated as percentages of their total number of bouts; **(b)** probability distribution (PDF) of the preferred speed for each bout duration category, including data from all subjects. First, the preferred speed of each subject for each bout duration was computed, then the PDF was estimated by the kernel smoothing function (*ksdensity* MATLAB); **(c)** boxplots of the preferred speed of different groups stratified by gender, age, BMI, and PA levels. The green line connects the median value of each group to highlight the underlying trend, (CoLaus cohort, Lausanne, Switzerland, 2014–2017).

Figure 2a shows the distribution (mean, SD) of gait bouts in different bout duration categories (i.e., < 30 s, [30–120] s, > 120 s, considered representative for indoor and outdoor walking), and for subjects stratified by age. Figure 2b illustrates the distribution of the preferred speed for each bout duration category, using the pooled data from all subjects. The effect of subject-specific factors (gender, age, BMI, PA level) on the preferred speed is graphically illustrated with the boxplots in Fig. 2c.

### Multivariable analysis: real-world gait speed, frailty and handgrip strength estimation

The results of logistic regression, conducted to assess the discrimination of frailty status, are shown in Table 2 and details are provided in Supplementary Tables S1–S3. As indicated by a lower AIC/BIC and a significant LR test, Model B (including speed metrics) provides a substantial improvement in distinguishing frailty condition. Compared to model A, the AUC, AIC, and BIC of model B were enhanced in all conditions where an average improvement of 3.1 (%), 3.1 (%), and 2.7 (%) are respectively observed for all speed metrics in all bout durations, with slightly higher improvement for the range [30–120] s. Furthermore, the LR test suggests the 95th percentile as the best speed metric (in all bout durations) for discrimination of frailty status.

**Table 2:** Effect of adding the speed metrics to predict frailty status, CoLaus cohort, Lausanne, Switzerland, 2014–2017.

Name	Duration (s)	Speed metrics	AUC	LR	*p*-value	AIC	BIC
Model A	Each duration	None	0.763	NaN	NaN	1497.4	1544.8
Model B	< 30	Mode	0.781	41.26	< 0.001	1458.1	1511.5
		Median	0.789	58.62	< 0.001	1440.8	1494.1
		Mean	0.793	71.06	< 0.001	1428.3	1481.7
		75th percentile	0.789	60.28	< 0.001	1439.1	1492.5
		90th percentile	0.796	72.72	< 0.001	1426.7	1480.0
		95th percentile	0.798	77.27	< 0.001	1422.1	1475.5
		Maximum	0.782	40.34	< 0.001	1459.1	1512.4
		Standard deviation	0.781	31.74	< 0.001	1467.6	1521.0
	30–120	Mode	0.781	41.46	< 0.001	1457.9	1511.3
		Median	0.788	57.59	< 0.001	1441.8	1495.2
		Mean	0.793	68.59	< 0.001	1430.8	1484.2
		75th percentile	0.789	59.28	< 0.001	1440.1	1493.5
		90th percentile	0.795	70.06	< 0.001	1429.3	1482.7
		95th percentile	0.800	78.04	< 0.001	1421.4	1474.7
		Maximum	0.785	43.70	< 0.001	1455.7	1509.1
		Standard deviation	0.779	26.89	< 0.001	1472.5	1525.9
	> 120	Mode	0.778	28.28	< 0.001	1471.1	1524.5
		Median	0.785	38.60	< 0.001	1460.8	1514.2
		Mean	0.790	49.92	< 0.001	1449.5	1502.8
		75th percentile	0.785	38.49	< 0.001	1460.9	1514.3
		90th percentile	0.791	50.77	< 0.001	1448.6	1502.0
		95th percentile	0.795	58.76	< 0.001	1440.6	1494.0
		Maximum	0.770	15.46	< 0.001	1483.9	1537.3
		Standard deviation	0.774	17.07	< 0.001	1482.3	1535.7

Model A includes gender, age, BMI, and PA; model B consists of all variables from model A plus the variable of interest (the speed metric specified in each row). Models A and B were compared by likelihood ratio (LR) test.

*GB* gait bout, *NaN* the values which were not possible to be computed, *AUC* area under the ROC curve, *AIC* Akaike’s information criterion, *BIC* Bayesian information criterion.

**Figure 3:**
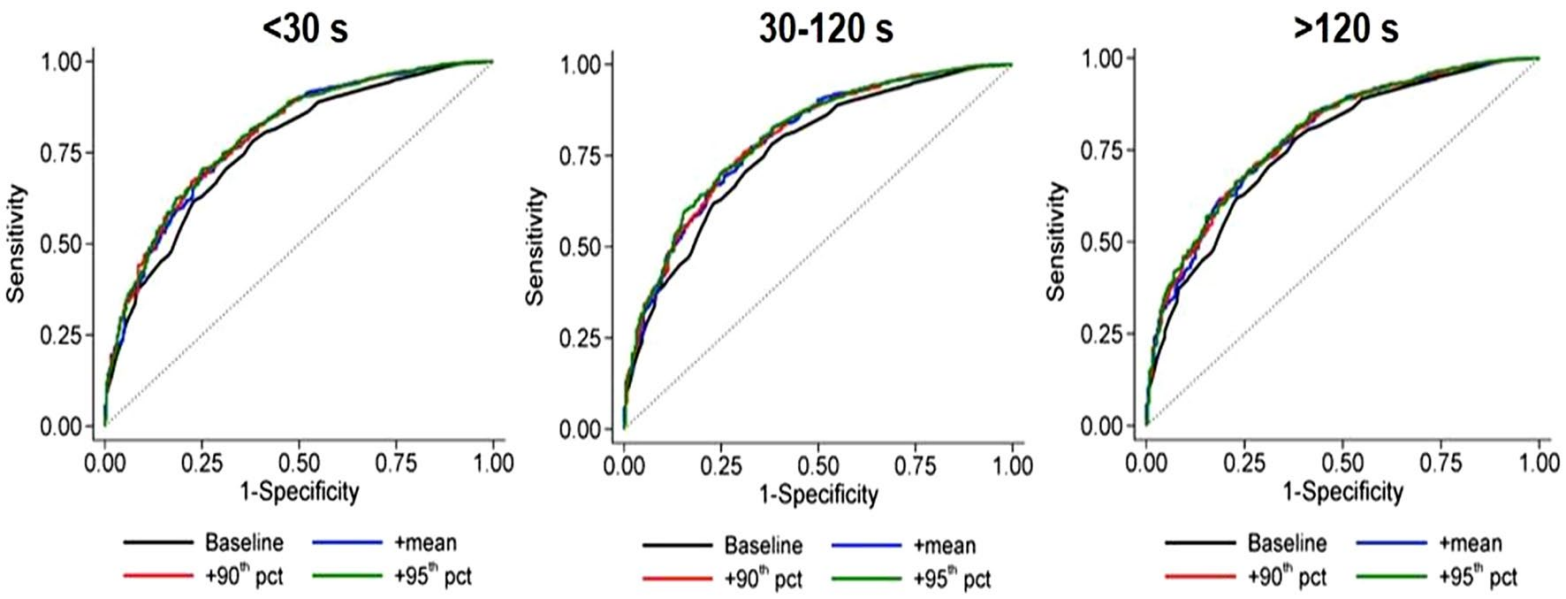
ROC of model A (baseline, i.e., without speed metrics) and B (baseline plus speed metrics: mean, + 90th, and + 95th percentile (pct)) in discrimination of frailty condition according to each bout duration, (CoLaus cohort, Lausanne, Switzerland, 2014–2017).

The results of linear regression for prediction of handgrip strength are shown in Table 3 and details are provided in Supplementary Tables S4–S6. Model B with inclusion of any speed metrics in all bout durations leads to a significant improvement compared to model A. Similarly, the 95th percentile of speed, and [30–120] s bout duration range showed more promising results. In order to visualize the difference between models A and B for discrimination of frailty, the ROC curves of both models are presented in Fig. 3. Here, the ROC curves for baseline (model A) and three speed metrics (mean, 90th, and 95th percentiles) are displayed for each bout duration category; it can be observed that the AUC of model A is lower than of model B.

**Table 3:** Effect of adding the speed metrics to estimate the handgrip strength, CoLaus cohort, Lausanne, Switzerland, 2014–2017.

Name	Duration (s)	Speed metrics	R^2^	LR	*p*-value	AIC	BIC
Model A	Each duration	None	0.648	NaN	NaN	18,780.5	18,827.9
Model B	< 30	Mode	0.660	100.91	< 0.001	18,681.6	18,734.9
		Median	0.673	210.80	< 0.001	18,571.7	18,625.0
		Mean	0.681	278.01	< 0.001	18,504.4	18,557.8
		75th percentile	0.676	234.44	< 0.001	18,548.0	18,601.4
		90th percentile	0.683	290.61	< 0.001	18,491.8	18,545.2
		95th percentile	0.685	309.43	< 0.001	18,473.0	18,526.4
		Maximum	0.662	112.55	< 0.001	18,669.9	18,723.3
		Standard deviation	0.664	135.30	< 0.001	18,647.2	18,700.5
	30–120	Mode	0.661	107.18	< 0.001	18,675.3	18,728.6
		Median	0.672	198.52	< 0.001	18,583.9	18,637.3
		Mean	0.679	257.77	< 0.001	18,524.7	18,578.1
		75th percentile	0.674	215.08	< 0.001	18,567.4	18,620.7
		90th percentile	0.679	260.00	< 0.001	18,522.5	18,575.8
		95th percentile	0.682	285.11	< 0.001	18,497.3	18,550.7
		Maximum	0.661	111.52	< 0.001	18,670.9	18,724.3
		Standard deviation	0.660	101.86	< 0.001	18,680.6	18,734.0
	> 120	Mode	0.659	91.90	< 0.001	18,690.6	18,743.9
		Median	0.669	170.34	< 0.001	18,612.1	18,665.5
		Mean	0.675	221.42	< 0.001	18,561.0	18,614.4
		75th percentile	0.667	159.97	< 0.001	18,622.5	18,675.9
		90th percentile	0.674	213.48	< 0.001	18,569.0	18,622.3
		95th percentile	0.674	220.24	< 0.001	18,562.2	18,615.6
		Maximum	0.656	66.16	< 0.001	18,716.3	18,769.7
		Standard deviation	0.654	53.03	< 0.001	18,729.4	18,782.8

Model A includes gender, age, BMI, and PA; model B consists of all variables from model A plus the variable of interest (the speed metric specified in each row). Models A and B were compared by likelihood ratio (LR) test.

*NaN* the values which were not possible to be computed, *AIC* Akaike’s information criterion, *BIC* Bayesian information criterion.

The results of stepwise regression models using different speed metrics within different bout duration categories in association with being non-frail and handgrip strength are presented in Table 4 and the details are provided in Supplementary Table S7. These results also support the previous results where the 95th percentile of speed distribution entered into the model for all bout durations.

**Table 4:** Summary of results of stepwise regression forward models for identification of non-frailty condition and prediction of handgrip strength, (CoLaus cohort, Lausanne, Switzerland, 2014–2017).

Speed metrics	*p*-values for Non-frailty			*p*-values for Handgrip strength		
	< 30 s	30–120 s	> 120 s	< 30 s	30–120 s	> 120 s
Mode	0.048	–	–	–	–	–
Median	–	–	–	–	–	–
Mean	–	–	–	–	–	< 0.001
75th percentile	–	–	–	–	–	< 0.001
90th percentile	–	–	–	–	–	–
95th percentile	< 0.001	< 0.001	< 0.001	< 0.001	< 0.001	< 0.001
Maximum	0.044	0.022	–	–	0.006	–
Standard deviation	–	0.018	–	< 0.001	< 0.001	–

*s* seconds.

‘–’The variable did not remain in the stepwise approach. The table reports the significant *p*-values obtained by using each speed metric within each bout duration category.

### Sensitivity analyses

Exclusion of participants with high speed (assumed as running) leads to excluding 753 out of 2809 subjects. The result of the repeated multivariable analysis for the remainder of participants (2056 subjects) can be found in the Supplementary Tables S8–S17. The exclusion of running data had no significant impact on the multivariable analyses results.

The results of the analyses conducted in participants aged 65 years and over are presented in Supplementary Tables S18–S27. Results were similar to those of the main analyses, i.e. all gait speed metrics improved the prediction of frailty and were significantly associated with handgrip strength. Further, and as in the main analysis, the 95th percentile was the variable most associated with both frailty and handgrip strength.

## Discussion

The results of this study demonstrated the feasibility of using a wrist acceleration-based method for long-term real-world monitoring of gait speed in a large cohort of community-dwelling adults. Cross-sectional univariate and multivariable analysis demonstrated that the distribution of preferred speed was consistent with established normative values published in literature, when subjects were stratified by demographic data such as gender, age, BMI, and daily PA levels^32,37^. It also highlights that the statistical speed metrics derived from the distribution of speed within different bout durations improved the discriminative power of regression models built for detection of frailty status, and prediction of muscle weakness/handgrip strength. The large sample size (*n* = 2809) and the uniform distributions of the subjects within each category of demographic data and PA levels allowed a comprehensive, valid statistical analysis of the gait speed metrics.

The range of preferred gait speed in our study (1–1.6 m/s), for each range of bout duration, is consistent with the normative values reported by several previous studies^32,37^ and recent systematic review/meta-analysis^38^. Interestingly, the trend observed with age and gender appeared similar to data recently reported on a big Japanese population-based cohort study, where outdoor gait speed was assessed using the smartphone-integrated GNSS technology^32^. Nevertheless, this technology may raise privacy issues and might not be accepted by the ethical committee in clinical research. Similarly, the percentage of the total number of gait bouts within each duration range (Fig. 2a) appeared similar to results reported in other studies^10,39,40^, indicating an exponential reduction of the number of bouts for longer durations. From Fig. 2a, it can be observed that about 65% were short (< 30 s), 30% were medium duration (30–120 s), and only 5% were long (> 120 s), and this trend was similar for all age categories. It was also observed that the longer bouts had a higher variation of speed. One possible explanation for this behavior would be the fact that the long bouts were related to outdoor activities, which are more influenced by contextual factors (e.g., quick utilitarian walk to go to the corner shop, or peaceful recreational walk in a park). An expected effect of gender, age, BMI, and PA levels on the preferred speed was observed. The trends in Fig. 2c are also consistent with the literature^6^ and show the importance of real-world gait speed for monitoring and assessing functional decline caused by factors such as aging and obesity.

Our study demonstrated that each of the speed metrics in the various bout durations improved the discrimination of the frailty status and handgrip strength. However, the improvements for medium duration bouts (30–120 s) appear slightly higher than the other periods. One explanation might be that short bouts (< 30 s) are usually related to indoor activity, such as stepping in a constrained environment during daily tasks. Hence, individuals are not necessarily challenged by their walking ability. On the other side, the long bouts (> 120 s) represent only a small percent of daily gait, so the limited number of samples available may decrease the statistical power. The medium bouts appear optimal for the estimation of robust speed metrics because their number is sufficiently high (about 30%), and the duration long enough to correspond to a stable gait pattern.

The 95th percentile appears as the speed metric with the stronger association with frailty condition. The significant difference between models A and B, and increased AUC for 95th percentile revealed that this speed metric, among other speed measurements, can serve the best as a proxy for the frailty condition. The explanation is that the 95th percentile is an estimation of the upper-bound of speed distribution, therefore it is more likely to reflect the physical/physiological reserve of the person. This result is in line with^37^ where a key observation was that maximum gait speed declines more steeply than comfortable gait speed with increasing age. Findings were similar when the analysis was restricted to participants aged 65 years and over, indicating that those metrics can be used in all age groups and to monitor disability in aging, as suggested recently^41^.

Real-world speed metrics were also associated with handgrip strength/muscle weakness. Clinically, these results were expected and in agreement with existing studies^37,42,43^. Indeed, a reduction of both gait speed and handgrip strength may appear due to muscle weakness, which is one of the cardinal signs of frailty/transition to frailty. A Canadian study on older adults also found that gait speed alone was sensitive and specific as a proxy for the Fried frailty phenotype. However, the dual-trait measure of gait speed with grip strength was more sensitive than individual traits and other possible dual-factor combinations^11^. A systematic review also concluded that, in predicting the risk of adverse outcomes, gait speed alone was as good as other composite tools^13^.

The main strengths of this study are the assessment of gait speed in a long-duration monitoring protocol (i.e., 13 successive days), in a large (*n* = 2809) and diverse cohort (i.e., both gender, and various age and BMI), and under entirely free-living situations using acceleration data from a wrist-worn device. The comprehensive statistical analyses on such a rich database led to a reliable understanding of the importance of real-world speed estimation in clinical applications. Furthermore, the methodology presented could help analyze massive existing databases recorded with GENEActiv device (http://mmarch.org/mmarch-sites)^44^, or other wrist-worn devices that record raw acceleration data^45^.

This study also has some limitations. First, it was conducted in a community-dwelling population that can be considered mostly healthy; hence, the results might not apply to hospitalized and more impaired people. Second, frailty was defined using a small set of criteria. There were an imbalanced number of subjects in the fit and frail groups; the results might change if the definitions based on other criteria would be used. Still, the definition of frailty used in this study is reliable in functional performance^33^. Finally, this study was cross-sectional where causality between PA and frailty or handgrip strength cannot be established; the ongoing follow-up of the CoLaus cohort will allow such assessment.

## Methods

### Study population

Subjects were enrolled in the framework of CoLaus|PsyCoLaus cohort study (https://www.colaus-psycolaus.ch/), an ongoing prospective survey investigating the biological and genetic determinants of cardiovascular risk factors and cardiovascular disease in the population of Lausanne, Switzerland^46^. A simple, non-stratified random sample of population aged between 35 and 75 years was drawn based on the following inclusion criteria: (i) written informed consent and (ii) willingness to take part in the examination and to provide blood samples. The baseline survey was conducted between 2003 and 2006, the first follow-up between 2009 and 2012, and the second follow-up between 2014 and 2017. The baseline and subsequent follow-ups included an interview, a physical exam, blood sampling, and questionnaires. In the second follow-up (4881 participants), daily PA was also assessed by accelerometry; hence, data from the second follow-up was used in cross-sectional analysis.

### Data collection

Participants have worn a lightweight (16 g), waterproof device including a 3-axial accelerometer (GENEActiv Original, ActivInsights Ltd, UK) on the wrist (dominant hand) for 13 successive full days in their free-living situations without any supervision or intervention. The acceleration was recorded with a range of ± 8 g and a sampling frequency of 50 Hz, where the accelerometer was calibrated based on^47^.

### Gait bouts detection, speed estimation and speed metrics

**Figure 4:**
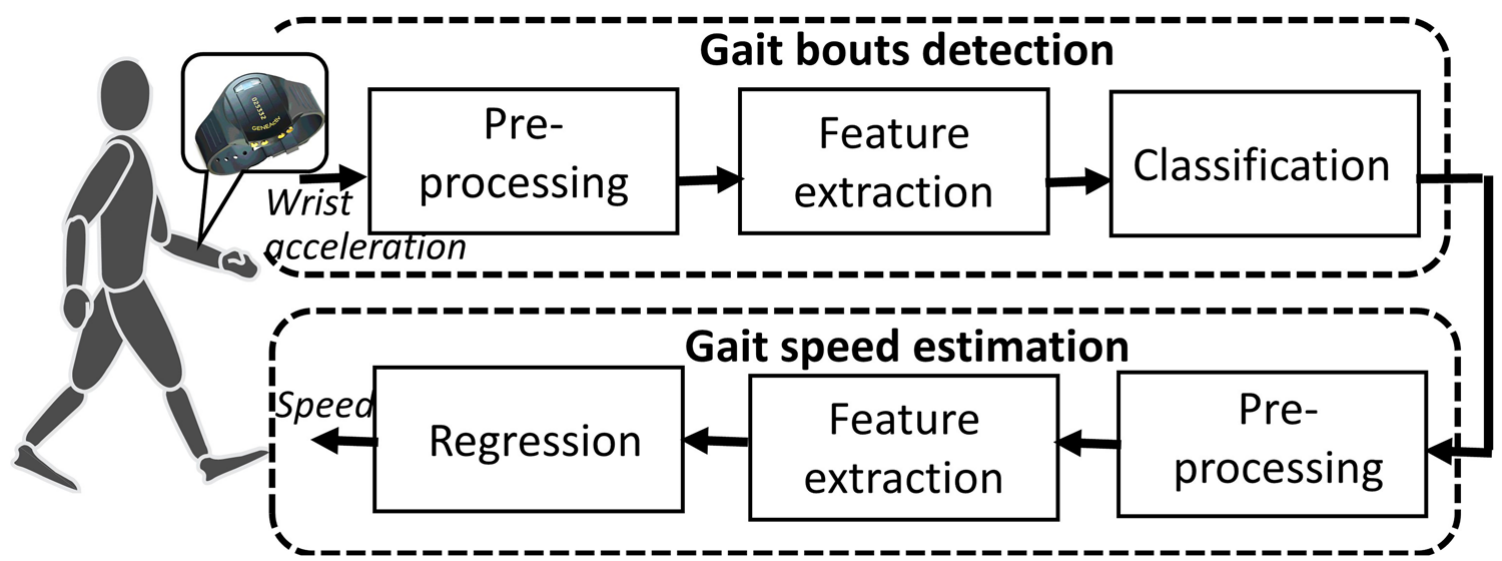
Block diagram of the system deployed for real-world gait speed estimation. Two validated algorithms were used, one for gait bout detection and the second for gait speed estimation.

After recording, raw acceleration data were transferred to a computer for analysis, to identify the gait bouts and estimate speed using validated algorithms, as shown in Fig. 4^26,27^.

#### Bouts detection^27^

The acceleration signal was first enhanced, and several biomechanically meaningful features (defined based on intensity, periodicity, posture, and noisiness) were extracted. Then, the features were fed into a classification procedure consisting of a Bayes classifier followed by two smart post-classification blocks. The output of this algorithm was per-second labels for continuous gait bouts. A median sensitivity, specificity, accuracy, and precision, 90.2, 97.2, 96.6, and 80.0 were reported (values in %, the highest possible value 100 corresponds to full agreement between the validated algorithm and the ground truth^48^).

#### Speed estimation^26^

The non-personalized version of the algorithm^26^ was used to estimate the speed of detected bouts. Relevant features such as energy, step frequency, mean of acceleration, intensity of wrist swing were extracted from the enhanced acceleration signal, and fed into a linear regression model to estimate the instantaneous speed (per second). A median root mean square error of 0.10 and 0.31 (m/s) were reported for the instantaneous walking and running speed estimation, respectively.

#### Speed metrics

For each subject, the detected bouts during monitoring time were stratified into three categories according to their durations (< 30 s; [30–120] s; > 120 s). The rationale was that short (< 30 s) bouts occur mostly indoors and are mainly context-dependent, whereas long (> 120 s) bouts happen mostly outdoors^49^. The other category ([30–120] s) could be a mixture of indoor and outdoor activities^49^. To characterize the ensemble of gait speed values, a set of metrics were derived from the statistical distribution of speed (average, mode, median, 75th, 90th, and 95th percentiles (pct), standard deviation (SD), and maximum) of each bout duration category.

All analyses were performed using MATLAB 2017b (MathWorks, USA).

### Other covariates

Age was categorized into four groups: [45–54], [55–64], [65–74] and [75 years. Body mass index (BMI) was categorized as normal (18.5 < BMI < 25 kg/m^2^), overweight (25 ≤ BMI < 30 kg/m^2^) and obese (≥ 30 kg/m^2^). As the percentage of underweight (BMI ≤ 18.5 kg/m^2^) participants was small (< 2%), they were included in the normal weight group.

Handgrip strength was assessed using the Baseline Hydraulic Hand Dynamometer (Enterprises Inc, Elmsford, NY, USA) with the subject seated, shoulders adducted and elbow flexed at 90°. Three measurements were performed consecutively with the right hand, and the highest value was included in the analyses. Frailty condition was defined using gender, BMI, and handgrip strength (kg) and categorized as frail and non-frail according to Fried et al.^33^, i.e., the frailty condition was identified as follows; for men: if BMI ≤ 24 and handgrip strength ≤ 29, or 24 < BMI ≤ 28 and handgrip strength ≤ 30, or BMI > 28 and handgrip strength ≤ 32; and for women: if BMI ≤ 23 and handgrip strength ≤ 17, or 23 < BMI ≤ 26 and handgrip strength ≤ 17.3, or 26 < BMI ≤ 29 and handgrip strength ≤ 18, or BMI > 29 and handgrip strength ≤ 21.

PA levels were estimated from the raw acceleration data using the R-package GGIR (http://cran.r-project.org)^47^ and the criteria of White et al. (https://github.com/Thomite/pampro/tree/v0.4.0) to define moderate and vigorous intensity. PA levels were further categorized into inactive (< 150 min/week) and active (≥ 150 min/week) of moderate and vigorous PA, respectively.

### Ethical statement

The Ethics Committee of the University of Lausanne, which afterward became the Ethics Commission of Canton Vaud (www.cer-vd.ch), approved the CoLaus study. The study was performed in agreement with the Helsinki declaration and its former amendments, and under the applicable Swiss legislation. All participants gave their signed informed consent before entering the study.

### Statistical analysis

Statistical analysis was performed using Stata 16 software (StataCorp. 2019. Stata Statistical Software: Release 16. College Station, TX: StataCorp LLC.) and MATLAB 2017b (MathWorks, USA). First, we performed univariate analysis to investigate the effect of each factor (gender, age, BMI, and PA levels) on gait speed, and to demonstrate the consistency between the speed values estimated in this study from the wrist-worn device and the normative/reference values in literature. Then, a comprehensive multivariable analysis was designed using the covariates and speed metrics as independent variables and the frailty index and handgrip strength as dependent variables.

#### Univariate analysis

For each subject the number of gait bouts in each duration category was calculated as percentage of total bouts detected, and the values were compared for subjects grouped by age categories. Then, the subject’s preferred/usual speed, for all bouts as well as for bouts in each duration category, was estimated as the mode (peak) of probability distribution function (PDF) using the Kernel Smoothing Function (*ksdensity*, MATLAB 2017b, MathWorks, USA). Besides, the preferred speed of subjects stratified by gender, age, BMI, and PA levels were compared through boxplots to highlight the effect of each factor.

#### Multivariable analysis

Two logistic regression nested models (A and B) were built to assess the importance of including gait speed for discrimination of frailty status. Model A included gender, age, BMI, and PA intensity levels, as these covariates are associated with frailty^33^. Model B used the same covariates as model A, plus the gait speed metrics. Similarly, two linear regression nested models (A and B) assessed the association with handgrip strength: model A included gender, age, BMI, and PA levels, and model B was the same as model A plus the gait speed metrics. For both analyses, the results were expressed as the area under the ROC (AUC) and Akaike’s and Bayesian information criteria (AIC and BIC, respectively). The improvement in detection of frailty, as well as the estimation of handgrip strength, was assessed by comparing model B with model A using likelihood ratio test (LR) and AUCs (for frailty) or adjusted R-square (for handgrip). The rationale of choosing nested models is that they can be compared statistically easily using the standard metrics (e.g., AIC, BIC, AUC). With this approach, the more complex one (Model B) was constructed by adding variable(s) (e.g., speed metrics) to the simpler one (Model A). To select the best out of these two models, we simply verified whether that added variable(s) explained a significant amount of additional variance in the data.

Eventually, to confirm the results of the previous analyses, a stepwise logistic regression (forward method) with a *p-*value for the entry of 0.05 was deployed to select the speed metric(s) with the stronger association with the non-frail condition. The same analysis was performed for handgrip strength with a stepwise linear regression. Due to the large number of tests performed, statistical significance was arbitrarily assessed for a two-sided test with *p* < 0.001.

#### Sensitivity analysis

The analysis algorithms^26,27^ could not distinguish between walking and running. Therefore, we excluded the participants whose 95th percentile of speed distribution (all bouts) was above the maximum value of walking speed reported in Ref.^37^. We then repeated the multivariable analysis as described above. We used the 95th percentile of speed since, statistically, it is more reliable than the maximum value. Finally, a second sensitivity analysis was conducted for participants aged 65 years and over.

Participation in the accelerometry study was voluntary, and a sizable fraction of the sample declined to participate. As this led to a non-random group with missing data, imputation of the missing data could not be performed^50^.

## Supplementary Information

Supplementary Information.
