# Supplementary material for: Real-world gait speed estimation, frailty and handgrip strength: a cohort-based study
Source: Sci Rep. 2021 Sep 23;11:18966. doi: 10.1038/s41598-021-98359-0 (PMC8460744; doi:10.1038/s41598-021-98359-0)
Supplement: Supplementary file 1 — Supplementary Information. [file 41598_2021_98359_MOESM1_ESM.docx]

**Supplementary files**

**Supplementary table S1**: full results to predict frailty, baseline model and models including the different metrics for bout periods <30 seconds, CoLaus cohort, Lausanne, Switzerland, 2014-2017.

|  | **Baseline** | **+ mode** | **+ median** | **+ mean** | **+ 75^th^ percentile** | **+ 90^th^ percentile** | **+ 95^th^ percentile** | **+ maximum** | **+ std deviation** |
| --- | --- | --- | --- | --- | --- | --- | --- | --- | --- |
| Man vs. woman | **0.51 (0.38-0.68)** | 0.74 (0.54-1.01) | 0.96 (0.69-1.34) | 1.18 (0.83-1.67) | 1.02 (0.73-1.43) | 1.28 (0.90-1.82) | 1.42 (0.98-2.06) | 0.81 (0.59-1.13) | 0.80 (0.58-1.11) |
| Age categories |  |  |  |  |  |  |  |  |  |
| [45-55[ | 1 (ref.) | 1 (ref.) | 1 (ref.) | 1 (ref.) | 1 (ref.) | 1 (ref.) | 1 (ref.) | 1 (ref.) | 1 (ref.) |
| [55-65[ | 1.50 (0.94-2.38) | 1.34 (0.83-2.14) | 1.32 (0.83-2.12) | 1.33 (0.83-2.13) | 1.38 (0.86-2.20) | 1.37 (0.86-2.19) | 1.36 (0.85-2.18) | 1.45 (0.91-2.32) | 1.47 (0.92-2.35) |
| [65-75[ | **2.66 (1.71-4.16)** | 2.13 (1.35-3.34) | 2.11 (1.34-3.32) | 2.13 (1.35-3.36) | **2.32 (1.47-3.64)** | **2.30 (1.46-3.62)** | 2.23 (1.41-3.50) | **2.39 (1.52-3.74)** | **2.59 (1.66-4.07)** |
| [75+ | **8.03 (5.08-12.7)** | **5.72 (3.57-9.17)** | **5.76 (3.59-9.22)** | **5.74 (3.59-9.20)** | **6.53 (4.09-10.4)** | **6.34 (3.97-10.1)** | **5.92 (3.70-9.47)** | **6.92 (4.35-11.0)** | **7.59 (4.78-12.1)** |
| BMI categories |  |  |  |  |  |  |  |  |  |
| Normal | 1 (ref.) | 1 (ref.) | 1 (ref.) | 1 (ref.) | 1 (ref.) | 1 (ref.) | 1 (ref.) | 1 (ref.) | 1 (ref.) |
| Overweight | 1.75 (1.24-2.47) | 1.65 (1.16-2.33) | 1.58 (1.11-2.23) | 1.54 (1.09-2.19) | 1.56 (1.10-2.21) | 1.52 (1.07-2.16) | 1.50 (1.06-2.13) | 1.59 (1.12-2.24) | 1.59 (1.12-2.25) |
| Obese | **3.79 (2.63-5.45)** | **3.52 (2.44-5.08)** | **3.38 (2.34-4.89)** | **3.21 (2.22-4.65)** | **3.31 (2.28-4.79)** | **3.06 (2.11-4.44)** | **2.98 (2.05-4.32)** | **3.29 (2.28-4.76)** | **3.25 (2.24-4.70)** |
| MVPA § | 0.63 (0.44-0.89) | 0.76 (0.53-1.08) | 0.77 (0.54-1.10) | 0.74 (0.52-1.06) | 0.74 (0.52-1.06) | 0.68 (0.48-0.97) | 0.64 (0.45-0.91) | 0.59 (0.42-0.84) | 0.61 (0.43-0.86) |
| Marker ‡ | - | **0.54 (0.45-0.66)** | **0.41 (0.32-0.52)** | **0.36 (0.28-0.46)** | **0.46 (0.37-0.56)** | **0.45 (0.38-0.55)** | **0.46 (0.38-0.55)** | **0.79 (0.73-0.85)** | **0.11 (0.05-0.25)** |
| AIC | 1497.4 | 1458.1 | 1440.8 | 1428.3 | 1439.1 | 1426.7 | 1422.1 | 1459.0 | 1467.6 |
| BIC | 1544.8 | 1511.5 | 1494.1 | 1481.7 | 1492.5 | 1480.0 | 1475.5 | 1512.4 | 1521.0 |
| AUC | 0.763 | 0.781 | 0.789 | 0.793 | 0.789 | 0.796 | 0.798 | 0.782 | 0.781 |
| LR-test † |  | 41.26 | 58.62 | 71.06 | 60.28 | 72.72 | 77.27 | 40.34 | 31.74 |
| p-value |  | <0.001 | <0.001 | <0.001 | <0.001 | <0.001 | <0.001 | <0.001 | <0.001 |

§ ≥150 vs. < 150 min/week; ‡ per 0.1 unit increase; †, compared to the baseline model. AIC, Akaike information criterion; AUC, area under the ROC; BIC, Bayesian information criterion. Results are expressed as odds-ratio and (95% confidence interval). Statistical analysis using logistic regression; significant results at P<0.001 are indicated in bold.

**Supplementary table S2**: full results to predict frailty, baseline model and models including the different metrics for bout periods between 30 and 120 seconds, CoLaus cohort, Lausanne, Switzerland, 2014-2017.

|  | **Baseline** | **+ mode** | **+ median** | **+ mean** | **+ 75^th^ percentile** | **+ 90^th^ percentile** | **+ 95^th^ percentile** | **+ maximum** | **+ std deviation** |
| --- | --- | --- | --- | --- | --- | --- | --- | --- | --- |
| Man vs. woman | **0.51 (0.38-0.68)** | 0.73 (0.54-0.99) | 0.94 (0.68-1.3) | 1.14 (0.81-1.6) | 1.01 (0.72-1.41) | 1.24 (0.87-1.76) | 1.41 (0.98-2.04) | 0.81 (0.58-1.11) | 0.76 (0.55-1.06) |
| Age categories |  |  |  |  |  |  |  |  |  |
| [45-55[ | 1 (ref.) | 1 (ref.) | 1 (ref.) | 1 (ref.) | 1 (ref.) | 1 (ref.) | 1 (ref.) | 1 (ref.) | 1 (ref.) |
| [55-65[ | 1.50 (0.94-2.38) | 1.35 (0.84-2.15) | 1.32 (0.82-2.11) | 1.33 (0.83-2.12) | 1.36 (0.85-2.17) | 1.39 (0.87-2.22) | 1.38 (0.86-2.21) | 1.46 (0.91-2.32) | 1.48 (0.93-2.36) |
| [65-75[ | **2.66 (1.71-4.16)** | 2.13 (1.36-3.36) | 2.12 (1.35-3.34) | 2.14 (1.36-3.37) | **2.31 (1.47-3.63)** | **2.35 (1.49-3.69)** | **2.27 (1.44-3.57)** | **2.41 (1.54-3.77)** | **2.62 (1.67-4.10)** |
| [75+ | **8.03 (5.08-12.7)** | **5.87 (3.66-9.39)** | **5.82 (3.64-9.31)** | **5.82 (3.64-9.31)** | **6.55 (4.1-10.45)** | **6.51 (4.07-10.4)** | **6.05 (3.79-9.67)** | **6.98 (4.39-11.1)** | **7.65 (4.82-12.2)** |
| BMI categories |  |  |  |  |  |  |  |  |  |
| Normal | 1 (ref.) | 1 (ref.) | 1 (ref.) | 1 (ref.) | 1 (ref.) | 1 (ref.) | 1 (ref.) | 1 (ref.) | 1 (ref.) |
| Overweight | 1.75 (1.24-2.47) | 1.63 (1.15-2.31) | 1.56 (1.10-2.22) | 1.53 (1.08-2.17) | 1.57 (1.10-2.22) | 1.51 (1.07-2.15) | 1.49 (1.05-2.11) | 1.62 (1.14-2.29) | 1.62 (1.15-2.30) |
| Obese | **3.79 (2.63-5.45)** | **3.52 (2.44-5.08)** | **3.33 (2.30-4.82)** | **3.18 (2.19-4.60)** | **3.30 (2.28-4.79)** | **3.06 (2.11-4.44)** | **2.96 (2.04-4.30)** | **3.37 (2.33-4.86)** | **3.33 (2.31-4.82)** |
| MVPA § | 0.63 (0.44-0.89) | 0.75 (0.53-1.06) | 0.77 (0.54-1.09) | 0.74 (0.52-1.06) | 0.74 (0.52-1.06) | 0.68 (0.48-0.97) | 0.65 (0.46-0.92) | 0.60 (0.42-0.84) | 0.61 (0.43-0.87) |
| Marker ‡ | - | **0.55 (0.45-0.66)** | **0.42 (0.34-0.53)** | **0.37 (0.29-0.48)** | **0.47 (0.38-0.57)** | **0.47 (0.39-0.56)** | **0.46 (0.39-0.55)** | **0.77 (0.71-0.84)** | **0.15 (0.07-0.31)** |
| AIC | 1497.4 | 1457.9 | 1441.8 | 1430.8 | 1440.1 | 1429.3 | 1421.3 | 1455.7 | 1472.5 |
| BIC | 1544.8 | 1511.3 | 1495.2 | 1484.2 | 1493.5 | 1482.7 | 1474.7 | 1509.0 | 1525.9 |
| AUC | 0.763 | 0.781 | 0.788 | 0.793 | 0.789 | 0.795 | 0.800 | 0.785 | 0.779 |
| LR-test † |  | 41.46 | 57.59 | 68.59 | 59.28 | 70.06 | 78.04 | 43.70 | 26.89 |
| p-value |  | <0.001 | <0.001 | <0.001 | <0.001 | <0.001 | <0.001 | <0.001 | <0.001 |

§ ≥150 vs. < 150 min/week; ‡ per 0.1 unit increase; †, compared to the baseline model. AIC, Akaike information criterion; AUC, area under the ROC; BIC, Bayesian information criterion. Results are expressed as odds-ratio and (95% confidence interval). Statistical analysis using logistic regression; significant results at P<0.001 are indicated in bold.

**Supplementary table S3**: full results to predict frailty, baseline model and models including the different metrics for bout periods above 120 seconds, CoLaus cohort, Lausanne, Switzerland, 2014-2017.

|  | **Baseline** | **+ mode** | **+ median** | **+ mean** | **+ 75^th^ percentile** | **+ 90^th^ percentile** | **+ 95^th^ percentile** | **+ maximum** | **+ std deviation** |
| --- | --- | --- | --- | --- | --- | --- | --- | --- | --- |
| Man vs. woman | **0.51 (0.38-0.68)** | **0.68 (0.50-0.93)** | 0.81 (0.59-1.11) | 0.94 (0.68-1.32) | 0.82 (0.59-1.13) | 0.98 (0.7-1.37) | 1.08 (0.76-1.53) | **0.67 (0.49-0.92)** | **0.66 (0.48-0.90)** |
| Age categories |  |  |  |  |  |  |  |  |  |
| [45-55[ | 1 (ref.) | 1 (ref.) | 1 (ref.) | 1 (ref.) | 1 (ref.) | 1 (ref.) | 1 (ref.) | 1 (ref.) | 1 (ref.) |
| [55-65[ | 1.50 (0.94-2.38) | 1.37 (0.86-2.18) | 1.37 (0.86-2.19) | 1.36 (0.85-2.18) | 1.42 (0.89-2.26) | 1.41 (0.88-2.26) | 1.42 (0.89-2.27) | 1.47 (0.92-2.34) | 1.50 (0.94-2.38) |
| [65-75[ | **2.66 (1.71-4.16)** | 2.23 (1.42-3.51) | **2.25 (1.43-3.54)** | **2.26 (1.44-3.55)** | **2.47 (1.57-3.87)** | **2.48 (1.58-3.89)** | **2.45 (1.56-3.84)** | **2.55 (1.63-3.99)** | **2.74 (1.75-4.29)** |
| [75+ | **8.03 (5.08-12.7)** | **6.45 (4.04-10.3)** | **6.40 (4.01-10.2)** | **6.23 (3.90-9.95)** | **6.96 (4.37-11.1)** | **6.72 (4.22-10.7)** | **6.42 (4.02-10.2)** | **7.21 (4.54-11.5)** | **7.78 (4.90-12.3)** |
| BMI categories |  |  |  |  |  |  |  |  |  |
| Normal | 1 (ref.) | 1 (ref.) | 1 (ref.) | 1 (ref.) | 1 (ref.) | 1 (ref.) | 1 (ref.) | 1 (ref.) | 1 (ref.) |
| Overweight | 1.75 (1.24-2.47) | 1.62 (1.15-2.29) | 1.59 (1.12-2.25) | 1.55 (1.10-2.20) | 1.58 (1.12-2.24) | 1.56 (1.10-2.21) | 1.54 (1.09-2.19) | 1.69 (1.19-2.38) | 1.65 (1.17-2.34) |
| Obese | **3.79 (2.63-5.45)** | **3.46 (2.40-4.99)** | **3.43 (2.38-4.95)** | **3.30 (2.29-4.78)** | **3.41 (2.36-4.92)** | **3.22 (2.22-4.66)** | **3.15 (2.18-4.57)** | **3.59 (2.49-5.17)** | **3.50 (2.43-5.05)** |
| MVPA § | 0.63 (0.44-0.89) | 0.71 (0.50-1.02) | 0.72 (0.50-1.02) | 0.71 (0.50-1.00) | 0.69 (0.49-0.98) | 0.65 (0.46-0.93) | 0.64 (0.45-0.91) | 0.64 (0.45-0.90) | 0.61 (0.43-0.87) |
| Marker ‡ | - | **0.64 (0.54-0.76)** | **0.54 (0.45-0.66)** | **0.48 (0.39-0.59)** | **0.60 (0.51-0.70)** | **0.58 (0.50-0.68)** | **0.57 (0.49-0.66)** | **0.85 (0.78-0.92)** | **0.32 (0.19-0.55)** |
| AIC | 1497.4 | 1471.1 | 1460.8 | 1449.5 | 1460.9 | 1448.6 | 1440.6 | 1483.9 | 1482.3 |
| BIC | 1544.8 | 1524.5 | 1514.1 | 1502.8 | 1514.3 | 1502.0 | 1494.0 | 1537.3 | 1535.7 |
| AUC | 0.763 | 0.778 | 0.785 | 0.790 | 0.785 | 0.791 | 0.795 | 0.770 | 0.774 |
| LR-test † |  | 28.28 | 38.60 | 49.92 | 38.49 | 50.77 | 58.76 | 15.46 | 17.07 |
| p-value |  | <0.001 | <0.001 | <0.001 | <0.001 | <0.001 | <0.001 | <0.001 | <0.001 |

§ ≥150 vs. < 150 min/week; ‡ per 0.1 unit increase; †, compared to the baseline model. AIC, Akaike information criterion; AUC, area under the ROC; BIC, Bayesian information criterion. Results are expressed as odds-ratio and (95% confidence interval). Statistical analysis using logistic regression; significant results at P<0.001 are indicated in bold.

**Supplementary table S4**: full results to predict handgrip, baseline model and models including the different metrics for bout periods <30 seconds, CoLaus cohort, Lausanne, Switzerland, 2014-2017.

|  | **Baseline** | **+ mode** | **+ median** | **+ mean** | **+ 75^th^ percentile** | **+ 90^th^ percentile** | **+ 95^th^ percentile** | **+ maximum** | **+ std deviation** |
| --- | --- | --- | --- | --- | --- | --- | --- | --- | --- |
| Man vs. woman | **17.7 (17.2; 18.3)** | **16.6 (16.1; 17.2)** | **15.4 (14.8; 16.0)** | **14.7 (14.0; 15.3)** | **15.2 (14.6; 15.8)** | **14.4 (13.8; 15.0)** | **14.1 (13.4; 14.7)** | **16.4 (15.8; 17.0)** | **16 (15.4; 16.6)** |
| Age categories |  |  |  |  |  |  |  |  |  |
| [45-55[ | 1 (ref.) | 1 (ref.) | 1 (ref.) | 1 (ref.) | 1 (ref.) | 1 (ref.) | 1 (ref.) | 1 (ref.) | 1 (ref.) |
| [55-65[ | **-3.0 (-3.6; -2.3)** | **-2.6 (-3.3; -1.9)** | **-2.5 (-3.1; -1.8)** | **-2.4 (-3.1; -1.8)** | **-2.6 (-3.2; -1.9)** | **-2.6 (-3.2; -1.9)** | **-2.5 (-3.2; -1.9)** | **-2.9 (-3.5; -2.2)** | **-2.9 (-3.6; -2.2)** |
| [65-75[ | **-6.2 (-7.0; -5.5)** | **-5.5 (-6.2; -4.7)** | **-5.3 (-6.0; -4.5)** | **-5.2 (-5.9; -4.5)** | **-5.6 (-6.3; -4.9)** | **-5.6 (-6.3; -4.9)** | **-5.4 (-6.1; -4.8)** | **-5.9 (-6.6; -5.2)** | **-6.2 (-6.9; -5.4)** |
| [75+ | **-9.9 (-10.9; -9.0)** | **-8.7 (-9.7; -7.8)** | **-8.4 (-9.3; -7.5)** | **-8.3 (-9.2; -7.4)** | **-8.8 (-9.7; -7.9)** | **-8.7 (-9.6; -7.8)** | **-8.4 (-9.3; -7.5)** | **-9.4 (-10.4; -8.5)** | **-9.6 (-10.5; -8.7)** |
| BMI categories |  |  |  |  |  |  |  |  |  |
| Normal | 1 (ref.) | 1 (ref.) | 1 (ref.) | 1 (ref.) | 1 (ref.) | 1 (ref.) | 1 (ref.) | 1 (ref.) | 1 (ref.) |
| Overweight | 0.6 (0; 1.2) | 0.8 (0.2; 1.4) | 1.0 (0.4; 1.6) | **1.1 (0.5; 1.7)** | **1.1 (0.5; 1.7)** | **1.2 (0.6; 1.8)** | **1.2 (0.7; 1.8)** | 0.8 (0.2; 1.4) | 1.0 (0.4; 1.6) |
| Obese | 0.4 (-0.4; 1.2) | 0.8 (0; 1.5) | 1.1 (0.3; 1.8) | 1.3 (0.5; 2.0) | 1.1 (0.4; 1.9) | **1.4 (0.7; 2.2)** | **1.5 (0.8; 2.3)** | 0.8 (0.1; 1.5) | 1.0 (0.3; 1.8) |
| MVPA § | 0.5 (-0.1; 1.1) | 0 (-0.6; 0.6) | -0.1 (-0.7; 0.4) | 0 (-0.5; 0.6) | 0 (-0.5; 0.6) | 0.3 (-0.2; 0.9) | 0.5 (-0.1; 1.0) | **0.7 (0.1; 1.3)** | **0.7 (0.1; 1.3)** |
| Marker ‡ |  | **1.7 (1.4; 2.0)** | **3.1 (2.7; 3.5)** | **3.7 (3.3; 4.1)** | **2.8 (2.5; 3.2)** | **2.8 (2.5; 3.1)** | **2.8 (2.5; 3.1)** | **0.7 (0.5; 0.8)** | **8.1 (6.7; 9.4)** |
| AIC | 18780.5 | 18681.6 | 18571.7 | 18504.4 | 18548.0 | 18491.8 | 18473.0 | 18669.9 | 18647.2 |
| BIC | 18827.9 | 18734.9 | 18625.0 | 18557.8 | 18601.4 | 18545.2 | 18526.4 | 18723.3 | 18700.5 |
| Adjusted R^2^ | 0.648 | 0.660 | 0.673 | 0.681 | 0.676 | 0.683 | 0.685 | 0.662 | 0.664 |
| LR-test † |  | 100.91 | 210.80 | 278.01 | 234.44 | 290.61 | 309.43 | 112.55 | 135.30 |
| p-value |  | <0.001 | <0.001 | <0.001 | <0.001 | <0.001 | <0.001 | <0.001 | <0.001 |

§ ≥150 vs. < 150 min/week; ‡ per 0.1 unit increase; †, compared to the baseline model. AIC, Akaike information criterion; BIC, Bayesian information criterion. Results are expressed as slope and (95% confidence interval). Statistical analysis using linear regression; significant results at P<0.001 are indicated in bold.

**Supplementary table S5**: full results to predict handgrip, baseline model and models including the different metrics for bout periods between 30 and 120 seconds, CoLaus cohort, Lausanne, Switzerland, 2014-2017.

|  | **Baseline** | **+ mode** | **+ median** | **+ mean** | **+ 75^th^ percentile** | **+ 90^th^ percentile** | **+ 95^th^ percentile** | **+ maximum** | **+ std deviation** |
| --- | --- | --- | --- | --- | --- | --- | --- | --- | --- |
| Man vs. woman | **17.7 (17.2; 18.3)** | **16.6 (16.1; 17.2)** | **15.6 (15.0; 16.2)** | **14.8 (14.2; 15.4)** | **15.3 (14.7; 15.9)** | **14.7 (14.0; 15.3)** | **14.3 (13.7; 14.9)** | **16.4 (15.9; 17.0)** | **16.3 (15.7; 16.9)** |
| Age categories |  |  |  |  |  |  |  |  |  |
| [45-55[ | 1 (ref.) | 1 (ref.) | 1 (ref.) | 1 (ref.) | 1 (ref.) | 1 (ref.) | 1 (ref.) | 1 (ref.) | 1 (ref.) |
| [55-65[ | **-3.0 (-3.6; -2.3)** | **-2.6 (-3.3; -1.9)** | **-2.5 (-3.1; -1.8)** | **-2.4 (-3.1; -1.8)** | **-2.6 (-3.2; -1.9)** | **-2.6 (-3.3; -2.0)** | **-2.6 (-3.2; -1.9)** | **-2.9 (-3.5; -2.2)** | **-2.9 (-3.6; -2.3)** |
| [65-75[ | **-6.2 (-7.0; -5.5)** | **-5.4 (-6.1; -4.7)** | **-5.3 (-6.0; -4.6)** | **-5.3 (-6.0; -4.6)** | **-5.6 (-6.3; -4.9)** | **-5.7 (-6.4; -5.0)** | **-5.6 (-6.3; -4.9)** | **-5.9 (-6.7; -5.2)** | **-6.2 (-6.9; -5.5)** |
| [75+ | **-9.9 (-10.9; -9.0)** | **-8.7 (-9.7; -7.8)** | **-8.5 (-9.4; -7.5)** | **-8.4 (-9.3; -7.5)** | **-8.9 (-9.8; -8.0)** | **-8.8 (-9.7; -7.9)** | **-8.6 (-9.5; -7.7)** | **-9.5 (-10.4; -8.6)** | **-9.7 (-10.6; -8.8)** |
| BMI categories |  |  |  |  |  |  |  |  |  |
| Normal | 1 (ref.) | 1 (ref.) | 1 (ref.) | 1 (ref.) | 1 (ref.) | 1 (ref.) | 1 (ref.) | 1 (ref.) | 1 (ref.) |
| Overweight | 0.6 (0; 1.2) | 0.8 (0.2; 1.4) | 1.0 (0.4; 1.6) | **1.1 (0.5; 1.7)** | **1.0 (0.5; 1.6)** | **1.2 (0.6; 1.7)** | **1.2 (0.7; 1.8)** | 0.8 (0.2; 1.3) | 0.9 (0.3; 1.5) |
| Obese | 0.4 (-0.4; 1.2) | 0.8 (0; 1.5) | 1.1 (0.3; 1.8) | 1.3 (0.5; 2.0) | 1.1 (0.3; 1.8) | **1.3 (0.6; 2.1)** | **1.5 (0.7; 2.2)** | 0.7 (0; 1.5) | 0.9 (0.1; 1.6) |
| MVPA § | 0.5 (-0.1; 1.1) | 0 (-0.5; 0.6) | -0.1 (-0.6; 0.5) | 0 (-0.6; 0.5) | 0.1 (-0.5; 0.6) | 0.3 (-0.2; 0.9) | 0.5 (-0.1; 1.0) | 0.6 (0.1; 1.2) | 0.7 (0.1; 1.2) |
| Marker ‡ |  | **1.7 (1.4; 2.1)** | **2.9 (2.5; 3.3)** | **3.5 (3.1; 3.9)** | **2.6 (2.3; 3.0)** | **2.6 (2.3; 2.9)** | **2.6 (2.3; 2.9)** | **0.7 (0.6; 0.8)** | **6.6 (5.3; 7.8)** |
| AIC | 18780.5 | 18675.3 | 18583.9 | 18524.7 | 18567.4 | 18522.5 | 18497.3 | 18670.9 | 18680.6 |
| BIC | 18827.9 | 18728.6 | 18637.3 | 18578.1 | 18620.7 | 18575.8 | 18550.7 | 18724.3 | 18734.0 |
| Adjusted R^2^ | 0.648 | 0.661 | 0.672 | 0.679 | 0.674 | 0.679 | 0.682 | 0.661 | 0.660 |
| LR-test † |  | 107.18 | 198.52 | 257.77 | 215.08 | 260.00 | 285.11 | 111.52 | 101.86 |
| p-value |  | <0.001 | <0.001 | <0.001 | <0.001 | <0.001 | <0.001 | <0.001 | <0.001 |

§ ≥150 vs. < 150 min/week; ‡ per 0.1 unit increase; †, compared to the baseline model. AIC, Akaike information criterion; BIC, Bayesian information criterion. Results are expressed as slope and (95% confidence interval). Statistical analysis using linear regression; significant results at P<0.001 are indicated in bold.

**Supplementary table S6**: full results to predict handgrip, baseline model and models including the different metrics for bout periods above 120 seconds, CoLaus cohort, Lausanne, Switzerland, 2014-2017.

|  | **Baseline** | **+ mode** | **+ median** | **+ mean** | **+ 75^th^ percentile** | **+ 90^th^ percentile** | **+ 95^th^ percentile** | **+ maximum** | **+ std deviation** |
| --- | --- | --- | --- | --- | --- | --- | --- | --- | --- |
| Man vs. woman | **17.7 (17.2; 18.3)** | **16.7 (16.2; 17.3)** | **15.9 (15.3; 16.5)** | **15.3 (14.7; 15.9)** | **15.9 (15.4; 16.5)** | **15.3 (14.7; 15.9)** | **15.1 (14.5; 15.7)** | **16.7 (16.1; 17.3)** | **16.9 (16.3; 17.5)** |
| Age categories |  |  |  |  |  |  |  |  |  |
| [45-55[ | 1 (ref.) | 1 (ref.) | 1 (ref.) | 1 (ref.) | 1 (ref.) | 1 (ref.) | 1 (ref.) | 1 (ref.) | 1 (ref.) |
| [55-65[ | **-3.0 (-3.6; -2.3)** | **-2.6 (-3.3; -2.0)** | **-2.6 (-3.2; -1.9)** | **-2.5 (-3.2; -1.9)** | **-2.7 (-3.3; -2.0)** | **-2.7 (-3.3; -2.0)** | **-2.7 (-3.3; -2.0)** | **-2.9 (-3.5; -2.2)** | **-2.9 (-3.6; -2.3)** |
| [65-75[ | **-6.2 (-7.0; -5.5)** | **-5.6 (-6.3; -4.8)** | **-5.5 (-6.2; -4.7)** | **-5.5 (-6.2; -4.8)** | **-5.8 (-6.5; -5.1)** | **-5.9 (-6.6; -5.2)** | **-5.8 (-6.5; -5.1)** | **-6.0 (-6.8; -5.3)** | **-6.3 (-7.0; -5.6)** |
| [75+ | **-9.9 (-10.9; -9.0)** | **-9.1 (-10; -8.1)** | **-8.8 (-9.7; -7.9)** | **-8.6 (-9.6; -7.7)** | **-9.2 (-10.1; -8.2)** | **-9.0 (-9.9; -8.1)** | **-8.9 (-9.8; -7.9)** | **-9.5 (-10.4; -8.5)** | **-9.8 (-10.7; -8.9)** |
| BMI categories |  |  |  |  |  |  |  |  |  |
| Normal | 1 (ref.) | 1 (ref.) | 1 (ref.) | 1 (ref.) | 1 (ref.) | 1 (ref.) | 1 (ref.) | 1 (ref.) | 1 (ref.) |
| Overweight | 0.6 (0; 1.2) | 0.8 (0.2; 1.4) | 0.9 (0.4; 1.5) | **1.0 (0.5; 1.6)** | 0.9 (0.4; 1.5) | 1.0 (0.4; 1.6) | **1.0 (0.5; 1.6)** | 0.7 (0.1; 1.3) | 0.8 (0.2; 1.3) |
| Obese | 0.4 (-0.4; 1.2) | 0.8 (0; 1.5) | 0.9 (0.2; 1.7) | 1.1 (0.4; 1.8) | 0.9 (0.1; 1.6) | 1.1 (0.4; 1.8) | 1.1 (0.4; 1.9) | 0.6 (-0.1; 1.4) | 0.6 (-0.1; 1.4) |
| MVPA § | 0.5 (-0.1; 1.1) | 0.1 (-0.5; 0.7) | 0.1 (-0.5; 0.6) | 0.1 (-0.4; 0.7) | 0.3 (-0.3; 0.8) | 0.4 (-0.1; 1.0) | 0.5 (-0.1; 1) | 0.5 (-0.1; 1.0) | **0.6 (0.1; 1.2)** |
| Marker ‡ |  | **1.4 (1.1; 1.7)** | **2.4 (2.0; 2.7)** | **2.9 (2.5; 3.2)** | **1.9 (1.6; 2.2)** | **2.0 (1.8; 2.3)** | **1.9 (1.7; 2.2)** | **0.6 (0.5; 0.7)** | **3.7 (2.7; 4.6)** |
| AIC | 18780.5 | 18690.6 | 18612.1 | 18561.0 | 18622.5 | 18569.0 | 18562.2 | 18716.3 | 18729.4 |
| BIC | 18827.9 | 18743.9 | 18665.5 | 18614.4 | 18675.9 | 18622.3 | 18615.6 | 18769.7 | 18782.8 |
| Adjusted R^2^ | 0.648 | 0.659 | 0.669 | 0.675 | 0.667 | 0.674 | 0.674 | 0.656 | 0.654 |
| LR-test † |  | 91.90 | 170.34 | 221.42 | 159.97 | 213.48 | 220.24 | 66.16 | 53.03 |
| p-value |  | <0.001 | <0.001 | <0.001 | <0.001 | <0.001 | <0.001 | <0.001 | <0.001 |

§ ≥150 vs. < 150 min/week; ‡ per 0.1 unit increase; †, compared to the baseline model. AIC, Akaike information criterion; BIC, Bayesian information criterion. Results are expressed as slope and (95% confidence interval). Statistical analysis using linear regression; significant results at P<0.001 are indicated in bold.

**Supplementary table S7**: stepwise analysis to predict frailty or handgrip, according to bout period, CoLaus cohort, Lausanne, Switzerland, 2014-2017.

|  |  | **Frailty** |  |  |  | **Handgrip** |  |
| --- | --- | --- | --- | --- | --- | --- | --- |
|  | **<30 seconds** | **30-120 seconds** | **>120 seconds** |  | **<30 seconds** | **30-120 seconds** | **>120 seconds** |
| Mode | 0.799 (0.639; 0.998) | - | - |  | - | - | - |
| Median | - | - | - |  | - | - | - |
| Mean | - | - | - |  | - | - | **3.09 (2.08; 4.10)** |
| 75^th^ percentile | - | - | - |  | - | - | **-1.45 (-2.22; -0.68)** |
| 90^th^ percentile | - | - | - |  | - | - | - |
| 95^th^ percentile | **0.568 (0.449; 0.719)** | **0.413 (0.312; 0.548)** | **0.572 (0.493; 0.663)** |  | **3.43 (2.95; 3.92)** | **3.14 (2.66; 3.62)** | **1.22 (0.79; 1.65)** |
| Maximum | 0.918 (0.844; 0.998) | 0.902 (0.826; 0.985) | - |  | - | 0.20 (0.06; 0.34) | - |
| Std deviation | - | 3.794 (1.257; 11.45) | - |  | -3.64 (-5.75; -1.53) | **-4.35 (-6.25; -2.44)** | - |

Results are expressed as odds ratio and (95% confidence interval) for frailty and slope and (95% confidence interval) for handgrip. Statistical analysis using logistic regression for frailty and linear regression for handgrip. -, not retained. Significant results at P<0.001 are indicated in bold.

**Supplementary table S8:** Summary of the results for detection of frailty after excluding participants with possible excessive speed (running), CoLaus cohort, Lausanne, Switzerland, 2014-2017.

| **Name** | **Duration (s)** | **Speed metrics** | **AUC** | **LR** | **p-value** | **AIC** | **BIC** |
| --- | --- | --- | --- | --- | --- | --- | --- |
| Model A | Each duration | None | 0.775 | NaN | NaN | 1099.1 | 1144.1 |
| Model B | < 30 |  |  |  |  |  |  |
|  |  | Mode | 0.783 | 23.74 | <0.001 | 1077.4 | 1127.9 |
|  |  | Median | 0.790 | 35.23 | <0.001 | 1065.9 | 1116.5 |
|  |  | Mean | 0.794 | 42.62 | <0.001 | 1058.5 | 1109.1 |
|  |  | 75^th^ percentile | 0.790 | 34.22 | <0.001 | 1066.9 | 1117.5 |
|  |  | 90^th^ percentile | 0.796 | 42.64 | <0.001 | 1058.5 | 1109.1 |
|  |  | 95^th^ percentile | 0.798 | 45.86 | <0.001 | 1055.3 | 1105.8 |
|  |  | Maximum | 0.783 | 20.75 | <0.001 | 1080.4 | 1130.9 |
|  |  | Standard deviation | 0.783 | 13.67 | <0.001 | 1087.5 | 1138.0 |
|  | 30-120 |  |  |  |  |  |  |
|  |  | Mode | 0.785 | 25.93 | <0.001 | 1075.2 | 1125.8 |
|  |  | Median | 0.789 | 33.50 | <0.001 | 1067.6 | 1118.2 |
|  |  | Mean | 0.794 | 40.95 | <0.001 | 1060.2 | 1110.7 |
|  |  | 75^th^ percentile | 0.789 | 32.07 | <0.001 | 1069.1 | 1119.6 |
|  |  | 90^th^ percentile | 0.795 | 38.99 | <0.001 | 1062.1 | 1112.7 |
|  |  | 95^th^ percentile | 0.800 | 45.94 | <0.001 | 1055.2 | 1105.8 |
|  |  | Maximum | 0.780 | 17.14 | <0.001 | 1084.0 | 1134.6 |
|  |  | Standard deviation | 0.780 | 8.89 | <0.001 | 1092.2 | 1142.8 |
|  | > 120 |  |  |  |  |  |  |
|  |  | Mode | 0.783 | 15.85 | <0.001 | 1085.3 | 1135.8 |
|  |  | Median | 0.788 | 23.50 | <0.001 | 1077.6 | 1128.2 |
|  |  | Mean | 0.792 | 30.29 | <0.001 | 1070.8 | 1121.4 |
|  |  | 75^th^ percentile | 0.787 | 22.49 | <0.001 | 1078.6 | 1129.2 |
|  |  | 90^th^ percentile | 0.792 | 29.71 | <0.001 | 1071.4 | 1122.0 |
|  |  | 95^th^ percentile | 0.795 | 34.71 | <0.001 | 1066.4 | 1117.0 |
|  |  | Maximum | 0.774 | 4.05 | <0.001 | 1097.1 | 1147.6 |
|  |  | Standard deviation | 0.778 | 6.23 | <0.001 | 1094.9 | 1145.5 |

AUC area under the ROC curve; NaN the values which were not possible to be computed; AIC, Akaike's information criterion; BIC, Bayesian information criterion. Model A includes gender, age, BMI, and PA; model B consists of all variables from model A plus the variable of interest (the speed metric specified in each row). Models A and B were compared by likelihood ratio (LR) test.

**Supplementary Table S9:** Summary of the results for estimating handgrip strength after excluding participants with possible excessive speed (running), CoLaus cohort, Lausanne, Switzerland, 2014-2017.

| **Name** | **Duration (s)** | **Speed metrics** | **LR** | **P-value** | **AIC** | **BIC** |
| --- | --- | --- | --- | --- | --- | --- |
| Model A | Each duration | None | NaN | NaN | 13851.4 | 13896.4 |
| Model B | < 30 |  |  |  |  |  |
|  |  | Mode | 49.44 | <0.001 | 13804.0 | 13854.5 |
|  |  | Median | 109.78 | <0.001 | 13743.6 | 13794.2 |
|  |  | Mean | 155.32 | <0.001 | 13698.1 | 13748.7 |
|  |  | 75^th^ percentile | 122.54 | <0.001 | 13730.9 | 13781.4 |
|  |  | 90^th^ percentile | 166.63 | <0.001 | 13686.8 | 13737.3 |
|  |  | 95^th^ percentile | 181.77 | <0.001 | 13671.6 | 13722.2 |
|  |  | Maximum | 61.10 | <0.001 | 13792.3 | 13842.9 |
|  |  | Standard deviation | 65.76 | <0.001 | 13787.7 | 13838.2 |
|  | 30-120 |  |  |  |  |  |
|  |  | Mode | 51.64 | <0.001 | 13801.8 | 13852.3 |
|  |  | Median | 99.48 | <0.001 | 13753.9 | 13804.5 |
|  |  | Mean | 140.31 | <0.001 | 13713.1 | 13763.7 |
|  |  | 75^th^ percentile | 107.41 | <0.001 | 13746.0 | 13796.6 |
|  |  | 90^th^ percentile | 143.68 | <0.001 | 13709.7 | 13760.3 |
|  |  | 95^th^ percentile | 169.80 | <0.001 | 13683.6 | 13734.2 |
|  |  | Maximum | 57.62 | <0.001 | 13795.8 | 13846.4 |
|  |  | Standard deviation | 43.69 | <0.001 | 13809.7 | 13860.3 |
|  | > 120 |  |  |  |  |  |
|  |  | Mode | 36.56 | <0.001 | 13816.9 | 13867.4 |
|  |  | Median | 77.23 | <0.001 | 13776.2 | 13826.7 |
|  |  | Mean | 108.33 | <0.001 | 13745.1 | 13795.6 |
|  |  | 75^th^ percentile | 66.44 | <0.001 | 13787.0 | 13837.5 |
|  |  | 90^th^ percentile | 100.52 | <0.001 | 13752.9 | 13803.5 |
|  |  | 95^th^ percentile | 118.98 | <0.001 | 13734.4 | 13785.0 |
|  |  | Maximum | 25.58 | <0.001 | 13827.8 | 13878.4 |
|  |  | Standard deviation | 13.64 | <0.001 | 13839.8 | 13890.3 |

NaN the values which were not possible to be computed; AIC, Akaike's information criterion; BIC, Bayesian information criterion. Model A includes gender, age categories, BMI categories, and PA; model B consists of all variables from model A plus the variable of interest (the speed metric specified in each row). Models A and B were compared by likelihood ratio (LR) test.

**Supplementary Table S10:** Summary of the re-analysis of the stepwise regression after excluding participants with possible excessive speed (running), CoLaus cohort, Lausanne, Switzerland, 2014-2017.

| **Speed metrics** | ***P*-values for frailty** | | | ***P*-values for Handgrip strength** | | |
| --- | --- | --- | --- | --- | --- | --- |
|  | **< 30 s** | **30-120 s** | **> 120 s** | **< 30 s** | **30-120 s** | **> 120 s** |
| Mode | - | - | - | - |  | - |
| Median | - | - | - | - |  | - |
| Mean | - | - | - | - |  | - |
| 75^th^ percentile | - | - | - | - |  | - |
| 90^th^ percentile | - | - | - | - |  | - |
| 95^th^ percentile | **<0.001** | **<0.001** | **<0.001** | **<0.001** | **<0.001** | **<0.001** |
| Maximum | - | - | - | - | - | - |
| Standard deviation | - | 0.016 | - | <0.004 | **<0.001** | **<0.001** |

s, seconds; '-' the variable was not retained in the stepwise approach. The table reports the *P*-values obtained by using each speed metric within each duration. *P*-values <0.001 are indicated in bold.

**Supplementary table S11**: full results to predict frailty, baseline model and models including the different metrics for bout periods <30 seconds, CoLaus cohort, Lausanne, Switzerland, 2014-2017. Analysis excluding participants with possible excessive speed (running).

|  | **Baseline** | **+ mode** | **+ median** | **+ mean** | **+ 75^th^ percentile** | **+ 90^th^ percentile** | **+ 95^th^ percentile** | **+ maximum** | **+ std deviation** |
| --- | --- | --- | --- | --- | --- | --- | --- | --- | --- |
| Man vs. woman | **0.41 (0.29-0.57)** | 0.62 (0.43-0.90) | 0.88 (0.58-1.33) | 1.14 (0.73-1.78) | 0.95 (0.62-1.45) | 1.26 (0.79-2.00) | 1.45 (0.89-2.35) | 0.68 (0.46-1.01) | 0.65 (0.43-0.98) |
| Age categories |  |  |  |  |  |  |  |  |  |
| [45-55[ | 1 (ref.) | 1 (ref.) | 1 (ref.) | 1 (ref.) | 1 (ref.) | 1 (ref.) | 1 (ref.) | 1 (ref.) | 1 (ref.) |
| [55-65[ | 1.79 (1.11-2.87) | 1.53 (0.95-2.47) | 1.44 (0.89-2.33) | 1.41 (0.87-2.28) | 1.48 (0.92-2.39) | 1.43 (0.88-2.31) | 1.40 (0.87-2.27) | 1.65 (1.02-2.65) | 1.64 (1.02-2.64) |
| [65-75[ | **3.89 (2.37-6.39)** | **2.75 (1.64-4.60)** | 2.34 (1.39-3.95) | 2.19 (1.30-3.69) | 2.47 (1.48-4.14) | 2.29 (1.37-3.83) | 2.15 (1.28-3.60) | 2.99 (1.80-4.97) | 3.17 (1.91-5.26) |
| [75+ | **10.4 (6.25-17.4)** | **6.83 (3.99-11.7)** | **6.11 (3.57-10.5)** | **5.75 (3.35-9.86)** | **6.69 (3.94-11.4)** | **6.15 (3.61-10.5)** | **5.68 (3.32-9.71)** | **8.21 (4.88-13.8)** | **8.64 (5.14-14.5)** |
| BMI categories |  |  |  |  |  |  |  |  |  |
| Normal | 1 (ref.) | 1 (ref.) | 1 (ref.) | 1 (ref.) | 1 (ref.) | 1 (ref.) | 1 (ref.) | 1 (ref.) | 1 (ref.) |
| Overweight | 1.68 (1.12-2.54) | 1.63 (1.08-2.47) | 1.61 (1.06-2.44) | 1.59 (1.05-2.42) | 1.60 (1.06-2.44) | 1.58 (1.04-2.40) | 1.55 (1.02-2.36) | 1.60 (1.06-2.42) | 1.61 (1.06-2.43) |
| Obese | **3.24 (2.10-4.99)** | **3.09 (2.01-4.78)** | **3.08 (1.99-4.76)** | **3.01 (1.94-4.67)** | **3.08 (1.99-4.78)** | **2.95 (1.90-4.58)** | **2.88 (1.85-4.47)** | **3.06 (1.98-4.72)** | **3.05 (1.97-4.71)** |
| MVPA § | 0.60 (0.40-0.89) | 0.72 (0.48-1.08) | 0.74 (0.49-1.11) | 0.71 (0.48-1.07) | 0.70 (0.47-1.05) | 0.64 (0.43-0.96) | 0.61 (0.41-0.91) | 0.57 (0.38-0.85) | 0.57 (0.38-0.85) |
| Marker ‡ | - | **0.57 (0.45-0.72)** | **0.42 (0.31-0.56)** | **0.36 (0.26-0.49)** | **0.47 (0.36-0.61)** | **0.45 (0.36-0.58)** | **0.45 (0.36-0.57)** | **0.81 (0.73-0.89)** | **0.17 (0.06-0.43)** |
| AIC | 1099.1 | 1077.4 | 1065.9 | 1058.5 | 1066.9 | 1058.5 | 1055.3 | 1080.4 | 1087.5 |
| BIC | 1144.1 | 1127.9 | 1116.5 | 1109.1 | 1117.5 | 1109.1 | 1105.8 | 1130.9 | 1138.0 |
| AUC | 0.775 | 0.783 | 0.790 | 0.794 | 0.790 | 0.796 | 0.798 | 0.783 | 0.783 |
| LR-test † |  | 23.74 | 35.23 | 42.62 | 34.22 | 42.64 | 45.86 | 20.75 | 13.67 |
| p-value |  | <0.001 | <0.001 | <0.001 | <0.001 | <0.001 | <0.001 | <0.001 | <0.001 |

§ ≥150 vs. < 150 min/week; ‡ per 0.1 unit increase; †, compared to the baseline model. AIC, Akaike information criterion; AUC, area under the ROC; BIC, Bayesian information criterion. Results are expressed as odds-ratio and (95% confidence interval). Statistical analysis using logistic regression; significant results at P<0.001 are indicated in bold.

**Supplementary table S12**: full results to predict frailty, baseline model and models including the different metrics for bout periods between 30 and 120 seconds, CoLaus cohort, Lausanne, Switzerland, 2014-2017. Analysis excluding participants with possible excessive speed (running).

|  | **Baseline** | **+ mode** | **+ median** | **+ mean** | **+ 75^th^ percentile** | **+ 90^th^ percentile** | **+ 95^th^ percentile** | **+ maximum** | **+ std deviation** |
| --- | --- | --- | --- | --- | --- | --- | --- | --- | --- |
| Man vs. woman | **0.41 (0.29-0.57)** | 0.63 (0.43-0.91) | 0.84 (0.56-1.26) | 1.08 (0.70-1.68) | 0.90 (0.59-1.38) | 1.17 (0.74-1.84) | 1.43 (0.88-2.31) | 0.63 (0.43-0.94) | 0.59 (0.39-0.88) |
| Age categories |  |  |  |  |  |  |  |  |  |
| [45-55[ | 1 (ref.) | 1 (ref.) | 1 (ref.) | 1 (ref.) | 1 (ref.) | 1 (ref.) | 1 (ref.) | 1 (ref.) | 1 (ref.) |
| [55-65[ | 1.79 (1.11-2.87) | 1.53 (0.95-2.47) | 1.45 (0.89-2.34) | 1.41 (0.87-2.28) | 1.47 (0.91-2.37) | 1.45 (0.90-2.35) | 1.42 (0.88-2.30) | 1.65 (1.02-2.65) | 1.67 (1.04-2.69) |
| [65-75[ | **3.89 (2.37-6.39)** | **2.70 (1.62-4.52)** | 2.39 (1.42-4.02) | 2.22 (1.32-3.74) | 2.50 (1.50-4.19) | 2.39 (1.43-3.98) | 2.19 (1.31-3.66) | 3.06 (1.84-5.08) | **3.31 (2.00-5.49)** |
| [75+ | **10.4 (6.25-17.4)** | **6.83 (4.00-11.7)** | **6.30 (3.68-10.8)** | **5.92 (3.46-10.1)** | **6.91 (4.07-11.7)** | **6.51 (3.83-11.0)** | **5.83 (3.42-9.94)** | **8.53 (5.07-14.4)** | **9.02 (5.38-15.2)** |
| BMI categories |  |  |  |  |  |  |  |  |  |
| Normal | 1 (ref.) | 1 (ref.) | 1 (ref.) | 1 (ref.) | 1 (ref.) | 1 (ref.) | 1 (ref.) | 1 (ref.) | 1 (ref.) |
| Overweight | 1.68 (1.12-2.54) | 1.63 (1.08-2.46) | 1.59 (1.05-2.42) | 1.57 (1.03-2.39) | 1.60 (1.05-2.43) | 1.57 (1.03-2.39) | 1.55 (1.02-2.36) | 1.66 (1.10-2.50) | 1.64 (1.09-2.48) |
| Obese | **3.24 (2.10-4.99)** | **3.10 (2.01-4.79)** | **3.04 (1.96-4.71)** | **2.97 (1.92-4.61)** | **3.09 (1.99-4.79)** | **2.95 (1.90-4.58)** | **2.87 (1.85-4.46)** | **3.16 (2.05-4.87)** | **3.10 (2.01-4.79)** |
| MVPA § | 0.60 (0.40-0.89) | 0.71 (0.47-1.06) | 0.73 (0.48-1.09) | 0.71 (0.48-1.07) | 0.70 (0.47-1.05) | 0.65 (0.43-0.97) | 0.61 (0.41-0.92) | 0.58 (0.39-0.86) | 0.58 (0.39-0.86) |
| Marker ‡ | - | **0.56 (0.45-0.71)** | **0.44 (0.33-0.58)** | **0.38 (0.28-0.51)** | **0.49 (0.38-0.63)** | **0.48 (0.38-0.61)** | **0.46 (0.37-0.58)** | **0.82 (0.74-0.91)** | 0.26 (0.11-0.63) |
| AIC | 1099.1 | 1075.2 | 1067.6 | 1060.2 | 1069.1 | 1062.1 | 1055.2 | 1084.0 | 1092.2 |
| BIC | 1144.1 | 1125.8 | 1118.2 | 1110.7 | 1119.6 | 1112.7 | 1105.8 | 1134.6 | 1142.8 |
| AUC | 0.775 | 0.785 | 0.789 | 0.794 | 0.789 | 0.795 | 0.800 | 0.780 | 0.780 |
| LR-test † |  | 25.93 | 33.5 | 40.95 | 32.07 | 38.99 | 45.94 | 17.14 | 8.89 |
| p-value |  | <0.001 | <0.001 | <0.001 | <0.001 | <0.001 | <0.001 | <0.001 | <0.001 |

§ ≥150 vs. < 150 min/week; ‡ per 0.1 unit increase; †, compared to the baseline model. AIC, Akaike information criterion; AUC, area under the ROC; BIC, Bayesian information criterion. Results are expressed as odds-ratio and (95% confidence interval). Statistical analysis using logistic regression; significant results at P<0.001 are indicated in bold.

**Supplementary table S13**: full results to predict frailty, baseline model and models including the different metrics for bout periods above 120 seconds, CoLaus cohort, Lausanne, Switzerland, 2014-2017. Analysis excluding participants with possible excessive speed (running).

|  | **Baseline** | **+ mode** | **+ median** | **+ mean** | **+ 75^th^ percentile** | **+ 90^th^ percentile** | **+ 95^th^ percentile** | **+ maximum** | **+ std deviation** |
| --- | --- | --- | --- | --- | --- | --- | --- | --- | --- |
| Man vs. woman | **0.41 (0.29-0.57)** | 0.57 (0.40-0.83) | 0.73 (0.49-1.08) | 0.89 (0.58-1.36) | 0.74 (0.49-1.10) | 0.94 (0.61-1.45) | 1.08 (0.69-1.69) | 0.5 (0.34-0.74) | 0.52 (0.36-0.76) |
| Age categories |  |  |  |  |  |  |  |  |  |
| [45-55[ | 1 (ref.) | 1 (ref.) | 1 (ref.) | 1 (ref.) | 1 (ref.) | 1 (ref.) | 1 (ref.) | 1 (ref.) | 1 (ref.) |
| [55-65[ | 1.79 (1.11-2.87) | 1.57 (0.97-2.53) | 1.52 (0.94-2.45) | 1.46 (0.90-2.36) | 1.55 (0.96-2.50) | 1.50 (0.93-2.42) | 1.48 (0.91-2.38) | 1.72 (1.07-2.76) | 1.71 (1.06-2.74) |
| [65-75[ | **3.89 (2.37-6.39)** | **2.91 (1.74-4.87)** | **2.64 (1.57-4.42)** | **2.44 (1.46-4.10)** | **2.83 (1.70-4.71)** | **2.63 (1.58-4.38)** | 2.47 (1.48-4.12) | **3.52 (2.12-5.82)** | **3.59 (2.18-5.91)** |
| [75+ | **10.4 (6.25-17.4)** | **7.61 (4.47-13.0)** | **7.01 (4.11-12.0)** | **6.51 (3.81-11.1)** | **7.54 (4.46-12.8)** | **6.93 (4.09-11.8)** | **6.43 (3.79-10.9)** | **9.27 (5.50-15.6)** | **9.44 (5.64-15.8)** |
| BMI categories |  |  |  |  |  |  |  |  |  |
| Normal | 1 (ref.) | 1 (ref.) | 1 (ref.) | 1 (ref.) | 1 (ref.) | 1 (ref.) | 1 (ref.) | 1 (ref.) | 1 (ref.) |
| Overweight | 1.68 (1.12-2.54) | 1.61 (1.07-2.44) | 1.59 (1.05-2.41) | 1.57 (1.03-2.38) | 1.58 (1.04-2.40) | 1.58 (1.04-2.39) | 1.59 (1.04-2.41) | 1.68 (1.11-2.53) | 1.65 (1.09-2.49) |
| Obese | **3.24 (2.10-4.99)** | **3.05 (1.97-4.70)** | **3.05 (1.97-4.71)** | **3.00 (1.94-4.65)** | **3.07 (1.99-4.75)** | **2.98 (1.93-4.62)** | **2.96 (1.91-4.60)** | **3.23 (2.10-4.98)** | **3.17 (2.06-4.90)** |
| MVPA § | 0.60 (0.40-0.89) | 0.67 (0.45-1.00) | 0.68 (0.45-1.01) | 0.67 (0.45-1.00) | 0.65 (0.44-0.97) | 0.62 (0.41-0.92) | 0.61 (0.41-0.90) | 0.61 (0.41-0.90) | 0.58 (0.39-0.87) |
| Marker ‡ | - | **0.66 (0.54-0.82)** | **0.55 (0.43-0.70)** | **0.47 (0.36-0.62)** | **0.60 (0.49-0.74)** | **0.57 (0.47-0.70)** | **0.55 (0.45-0.67)** | 0.90 (0.81-1.00) | 0.42 (0.21-0.83) |
| AIC | 1099.1 | 1085.3 | 1077.6 | 1070.8 | 1078.6 | 1071.4 | 1066.4 | 1097.1 | 1094.9 |
| BIC | 1144.1 | 1135.8 | 1128.2 | 1121.4 | 1129.2 | 1122.0 | 1117.0 | 1147.6 | 1145.5 |
| AUC | 0.775 | 0.783 | 0.788 | 0.792 | 0.787 | 0.792 | 0.795 | 0.774 | 0.778 |
| LR-test † |  | 15.85 | 23.50 | 30.29 | 22.49 | 29.71 | 34.71 | 4.05 | 6.23 |
| p-value |  | <0.001 | <0.001 | <0.001 | <0.001 | <0.001 | <0.001 | <0.001 | <0.001 |

§ ≥150 vs. < 150 min/week; ‡ per 0.1 unit increase; †, compared to the baseline model. AIC, Akaike information criterion; AUC, area under the ROC; BIC, Bayesian information criterion. Results are expressed as odds-ratio and (95% confidence interval). Statistical analysis using logistic regression; significant results at P<0.001 are indicated in bold.

**Supplementary table S14**: full results to predict handgrip, baseline model and models including the different metrics for bout periods <30 seconds, CoLaus cohort, Lausanne, Switzerland, 2014-2017. Analysis excluding participants with possible excessive speed (running).

|  | **Baseline** | **+ mode** | **+ median** | **+ mean** | **+ 75^th^ percentile** | **+ 90^th^ percentile** | **+ 95^th^ percentile** | **+ maximum** | **+ std deviation** |
| --- | --- | --- | --- | --- | --- | --- | --- | --- | --- |
| Man vs. woman | **18.3 (17.6; 18.9)** | **17.2 (16.5; 17.9)** | **15.9 (15.1; 16.7)** | **14.9 (14.1; 15.7)** | **15.6 (14.8; 16.4)** | **14.6 (13.7; 15.4)** | **14.1 (13.3; 15)** | **16.9 (16.2; 17.6)** | **16.6 (15.9; 17.4)** |
| Age categories |  |  |  |  |  |  |  |  |  |
| [45-55[ | 1 (ref.) | 1 (ref.) | 1 (ref.) | 1 (ref.) | 1 (ref.) | 1 (ref.) | 1 (ref.) | 1 (ref.) | 1 (ref.) |
| [55-65[ | **-3.8 (-4.6; -3.1)** | **-3.3 (-4.1; -2.6)** | **-2.9 (-3.6; -2.1)** | **-2.6 (-3.3; -1.8)** | **-2.9 (-3.6; -2.1)** | **-2.6 (-3.3; -1.9)** | **-2.5 (-3.2; -1.7)** | **-3.5 (-4.3; -2.8)** | **-3.3 (-4.1; -2.6)** |
| [65-75[ | **-7.8 (-8.7; -6.8)** | **-6.7 (-7.7; -5.7)** | **-5.9 (-6.9; -4.9)** | **-5.4 (-6.4; -4.4)** | **-6 (-6.9; -5.0)** | **-5.5 (-6.5; -4.6)** | **-5.3 (-6.2; -4.3)** | **-7 (-7.9; -6)** | **-6.9 (-7.9; -6.0)** |
| [75+ | **-11.5(-12.6;-10.3)** | **-10.2(-11.4;-9.0)** | **-9.5 (-10.7; -8.3)** | **-9.1 (-10.3; -7.9)** | **-9.7 (-10.9; -8.6)** | **-9.3 (-10.5; -8.2)** | **-9.0 (-10.2; -7.9)** | **-10.8(-11.9;-9.6)** | **-10.7(-11.9;-9.6)** |
| BMI categories |  |  |  |  |  |  |  |  |  |
| Normal | 1 (ref.) | 1 (ref.) | 1 (ref.) | 1 (ref.) | 1 (ref.) | 1 (ref.) | 1 (ref.) | 1 (ref.) | 1 (ref.) |
| Overweight | 0.7 (0; 1.4) | 0.8 (0.1; 1.5) | 0.9 (0.2; 1.6) | 1.0 (0.3; 1.7) | 1.0 (0.3; 1.7) | 1.1 (0.4; 1.8) | 1.2 (0.5; 1.9) | 0.9 (0.2; 1.6) | 1.0 (0.3; 1.7) |
| Obese | 0.5 (-0.4; 1.4) | 0.8 (-0.1; 1.6) | 1.0 (0.1; 1.8) | 1.1 (0.2; 2.0) | 1.0 (0.1; 1.9) | 1.2 (0.3; 2.0) | 1.3 (0.4; 2.1) | 0.7 (-0.2; 1.6) | 0.8 (0; 1.7) |
| MVPA § | 0.5 (-0.2; 1.2) | 0 (-0.7; 0.7) | -0.1 (-0.8; 0.5) | 0 (-0.7; 0.6) | 0.1 (-0.6; 0.7) | 0.3 (-0.3; 1.0) | 0.5 (-0.2; 1.1) | 0.6 (-0.1; 1.3) | 0.7 (0.1; 1.4) |
| Marker ‡ | - | **1.5 (1.1; 1.9)** | **2.9 (2.4; 3.5)** | **3.7 (3.1; 4.2)** | **2.7 (2.2; 3.2)** | **2.9 (2.4; 3.3)** | **2.9 (2.5; 3.3)** | **0.6 (0.5; 0.8)** | **7.3 (5.5; 9.0)** |
| AIC | 13851.4 | 13804.0 | 13743.6 | 13698.1 | 13730.9 | 13686.8 | 13671.6 | 13792.3 | 13787.7 |
| BIC | 13896.4 | 13854.5 | 13794.2 | 13748.7 | 13781.4 | 13737.3 | 13722.2 | 13842.9 | 13838.2 |
| Adjusted R^2^ | 0.628 | 0.637 | 0.647 | 0.655 | 0.649 | 0.657 | 0.659 | 0.639 | 0.640 |
| LR-test † |  | 49.44 | 109.78 | 155.32 | 122.54 | 166.63 | 181.77 | 61.10 | 65.76 |
| p-value |  | <0.001 | <0.001 | <0.001 | <0.001 | <0.001 | <0.001 | <0.001 | <0.001 |

§ ≥150 vs. < 150 min/week; ‡ per 0.1 unit increase; †, compared to the baseline model. AIC, Akaike information criterion; BIC, Bayesian information criterion. Results are expressed as slope and (95% confidence interval). Statistical analysis using linear regression; significant results at P<0.001 are indicated in bold.

**Supplementary table S15**: full results to predict handgrip, baseline model and models including the different metrics for bout periods between 30 and 120 seconds, CoLaus cohort, Lausanne, Switzerland, 2014-2017. Analysis excluding participants with possible excessive speed (running).

|  | **Baseline** | **+ mode** | **+ median** | **+ mean** | **+ 75^th^ percentile** | **+ 90^th^ percentile** | **+ 95^th^ percentile** | **+ maximum** | **+ std deviation** |
| --- | --- | --- | --- | --- | --- | --- | --- | --- | --- |
| Man vs. woman | **18.3 (17.6; 18.9)** | **17.2 (16.5; 17.9)** | **16.1 (15.3; 16.8)** | **15.2 (14.4; 16)** | **15.8 (15.1; 16.6)** | **15 (14.1; 15.8)** | **14.4 (13.5; 15.2)** | **16.9 (16.2; 17.6)** | **17.0 (16.2; 17.7)** |
| Age categories |  |  |  |  |  |  |  |  |  |
| [45-55[ | 1 (ref.) | 1 (ref.) | 1 (ref.) | 1 (ref.) | 1 (ref.) | 1 (ref.) | 1 (ref.) | 1 (ref.) | 1 (ref.) |
| [55-65[ | **-3.8 (-4.6; -3.1)** | **-3.3 (-4.1; -2.5)** | **-2.9 (-3.6; -2.1)** | **-2.6 (-3.4; -1.9)** | **-2.9 (-3.6; -2.1)** | **-2.7 (-3.4; -1.9)** | **-2.5 (-3.3; -1.8)** | **-3.4 (-4.2; -2.7)** | **-3.5 (-4.2; -2.7)** |
| [65-75[ | **-7.8 (-8.7; -6.8)** | **-6.6 (-7.6; -5.7)** | **-6.0 (-7.0; -5.0)** | **-5.6 (-6.5; -4.6)** | **-6.1 (-7.1; -5.1)** | **-5.8 (-6.8; -4.8)** | **-5.4 (-6.4; -4.5)** | **-6.9 (-7.9; -6.0)** | **-7.1 (-8.1; -6.2)** |
| [75+ | **-11.5(-12.6;-10.3)** | **-10.2 (-11.4; -9)** | **-9.6 (-10.8; -8.4)** | **-9.3 (-10.5; -8.1)** | **-9.9 (-11.1; -8.8)** | **-9.6 (-10.8; -8.5)** | **-9.2 (-10.4; -8.0)** | **-10.8(-11.9;-9.6)** | **-10.9(-12.1;-9.8)** |
| BMI categories |  |  |  |  |  |  |  |  |  |
| Normal | 1 (ref.) | 1 (ref.) | 1 (ref.) | 1 (ref.) | 1 (ref.) | 1 (ref.) | 1 (ref.) | 1 (ref.) | 1 (ref.) |
| Overweight | 0.7 (0; 1.4) | 0.8 (0.1; 1.5) | 1.0 (0.3; 1.7) | 1.1 (0.4; 1.8) | 1.0 (0.3; 1.7) | 1.1 (0.4; 1.8) | 1.2 (0.5; 1.9) | 0.8 (0.1; 1.5) | 0.9 (0.1; 1.6) |
| Obese | 0.5 (-0.4; 1.4) | 0.8 (-0.1; 1.7) | 1.0 (0.1; 1.8) | 1.1 (0.2; 2.0) | 0.9 (0; 1.8) | 1.1 (0.2; 2.0) | 1.2 (0.3; 2.1) | 0.6 (-0.3; 1.5) | 0.7 (-0.2; 1.6) |
| MVPA § | 0.5 (-0.2; 1.2) | 0 (-0.6; 0.7) | -0.1 (-0.7; 0.6) | 0 (-0.7; 0.6) | 0.1 (-0.6; 0.8) | 0.3 (-0.3; 1.0) | 0.5 (-0.2; 1.1) | 0.5 (-0.1; 1.2) | 0.7 (0; 1.3) |
| Marker ‡ | - | **1.5 (1.1; 1.9)** | **2.7 (2.2; 3.2)** | **3.4 (2.8; 4.0)** | **2.4 (2.0; 2.9)** | **2.6 (2.2; 3.0)** | **2.7 (2.3; 3.1)** | **0.6 (0.5; 0.8)** | **5.6 (3.9; 7.2)** |
| AIC | 13851.4 | 13801.8 | 13753.9 | 13713.1 | 13746.0 | 13709.7 | 13683.6 | 13795.8 | 13809.7 |
| BIC | 13896.4 | 13852.3 | 13804.5 | 13763.7 | 13796.6 | 13760.3 | 13734.2 | 13846.4 | 13860.3 |
| Adjusted R^2^ | 0.628 | 0.637 | 0.645 | 0.652 | 0.647 | 0.653 | 0.657 | 0.638 | 0.636 |
| LR-test † |  | 51.64 | 99.48 | 140.31 | 107.41 | 143.68 | 169.8 | 57.62 | 43.69 |
| p-value |  | <0.001 | <0.001 | <0.001 | <0.001 | <0.001 | <0.001 | <0.001 | <0.001 |

§ ≥150 vs. < 150 min/week; ‡ per 0.1 unit increase; †, compared to the baseline model. AIC, Akaike information criterion; BIC, Bayesian information criterion. Results are expressed as slope and (95% confidence interval). Statistical analysis using linear regression; significant results are indicated in bold.

**Supplementary table S16**: full results to predict handgrip, baseline model and models including the different metrics for bout periods above 120 seconds, CoLaus cohort, Lausanne, Switzerland, 2014-2017. Analysis excluding participants with possible excessive speed (running).

|  | **Baseline** | **+ mode** | **+ median** | **+ mean** | **+ 75^th^ percentile** | **+ 90^th^ percentile** | **+ 95^th^ percentile** | **+ maximum** | **+ std deviation** |
| --- | --- | --- | --- | --- | --- | --- | --- | --- | --- |
| Man vs. woman | **18.3 (17.6; 18.9)** | **17.4 (16.7; 18.1)** | **16.5 (15.8; 17.3)** | **15.8 (15.0; 16.6)** | **16.6 (15.9; 17.4)** | **15.8 (15.0; 16.6)** | **15.3 (14.5; 16.2)** | **17.4 (16.6; 18.1)** | **17.7 (17; 18.4)** |
| Age categories |  |  |  |  |  |  |  |  |  |
| [45-55[ | 1 (ref.) | 1 (ref.) | 1 (ref.) | 1 (ref.) | 1 (ref.) | 1 (ref.) | 1 (ref.) | 1 (ref.) | 1 (ref.) |
| [55-65[ | **-3.8 (-4.6; -3.1)** | **-3.4 (-4.1; -2.6)** | **-3.1 (-3.8; -2.3)** | **-2.8 (-3.6; -2.1)** | **-3.2 (-3.9; -2.4)** | **-2.9 (-3.7; -2.2)** | **-2.8 (-3.5; -2.0)** | **-3.6 (-4.3; -2.8)** | **-3.6 (-4.4; -2.9)** |
| [65-75[ | **-7.8 (-8.7; -6.8)** | **-6.9 (-7.9; -5.9)** | **-6.3 (-7.3; -5.3)** | **-5.9 (-6.9; -5.0)** | **-6.6 (-7.6; -5.7)** | **-6.3 (-7.2; -5.3)** | **-6.0 (-7.0; -5.0)** | **-7.3 (-8.2; -6.3)** | **-7.5 (-8.5; -6.6)** |
| [75+ | **-11.5(-12.6;-10.3)** | **-10.5(-11.7;-9.4)** | **-10 (-11.2; -8.8)** | **-9.7 (-10.9; -8.5)** | **-10.4(-11.5;-9.2)** | **-10 (-11.2; -8.8)** | **-9.7 (-10.8; -8.5)** | **-10.9(-12.1;-9.8)** | **-11.2(-12.4;-10.1)** |
| BMI categories |  |  |  |  |  |  |  |  |  |
| Normal | 1 (ref.) | 1 (ref.) | 1 (ref.) | 1 (ref.) | 1 (ref.) | 1 (ref.) | 1 (ref.) | 1 (ref.) | 1 (ref.) |
| Overweight | 0.7 (0; 1.4) | 0.8 (0.1; 1.5) | 0.9 (0.2; 1.6) | 1.0 (0.3; 1.7) | 0.9 (0.2; 1.6) | 1.0 (0.3; 1.7) | 1.0 (0.3; 1.7) | 0.8 (0; 1.5) | 0.8 (0.1; 1.5) |
| Obese | 0.5 (-0.4; 1.4) | 0.8 (-0.1; 1.6) | 0.9 (0; 1.8) | 1.0 (0.1; 1.8) | 0.8 (-0.1; 1.7) | 0.9 (0; 1.8) | 1.0 (0.1; 1.9) | 0.6 (-0.3; 1.5) | 0.6 (-0.3; 1.5) |
| MVPA § | 0.5 (-0.2; 1.2) | 0.2 (-0.5; 0.8) | 0.1 (-0.6; 0.8) | 0.2 (-0.5; 0.8) | 0.3 (-0.4; 1.0) | 0.5 (-0.2; 1.1) | 0.5 (-0.2; 1.1) | 0.4 (-0.3; 1.1) | 0.6 (-0.1; 1.2) |
| Marker ‡ | - | **1.1 (0.8; 1.5)** | **2.0 (1.6; 2.5)** | **2.6 (2.2; 3.1)** | **1.6 (1.2; 2.0)** | **1.9 (1.5; 2.3)** | **2.0 (1.7; 2.4)** | **0.5 (0.3; 0.7)** | **2.4 (1.1; 3.6)** |
| AIC | 13851.4 | 13816.9 | 13776.2 | 13745.1 | 13787.0 | 13752.9 | 13734.4 | 13827.8 | 13839.8 |
| BIC | 13896.4 | 13867.4 | 13826.7 | 13795.6 | 13837.5 | 13803.5 | 13785.0 | 13878.4 | 13890.3 |
| Adjusted R^2^ | 0.628 | 0.634 | 0.642 | 0.647 | 0.640 | 0.646 | 0.649 | 0.632 | 0.630 |
| LR-test † |  | 36.56 | 77.23 | 108.33 | 66.44 | 100.52 | 118.98 | 25.58 | 13.64 |
| p-value |  | <0.001 | <0.001 | <0.001 | <0.001 | <0.001 | <0.001 | <0.001 | <0.001 |

§ ≥150 vs. < 150 min/week; ‡ per 0.1 unit increase; †, compared to the baseline model. AIC, Akaike information criterion; BIC, Bayesian information criterion. Results are expressed as slope and (95% confidence interval). Statistical analysis using linear regression; significant results at P<0.001 are indicated in bold.

**Supplementary table S17**: stepwise analysis to predict frailty or handgrip, according to bout period, CoLaus cohort, Lausanne, Switzerland, 2014-2017. Analysis excluding participants with possible excessive speed (running).

|  |  | **Frailty** |  |  |  | **Handgrip** |  |
| --- | --- | --- | --- | --- | --- | --- | --- |
|  | **<30 seconds** | **30-120 seconds** | **>120 seconds** |  | **<30 seconds** | **30-120 seconds** | **>120 seconds** |
| Mode | - | - | - |  | - | - | - |
| Median | - | - | - |  | - | - | - |
| Mean | - | - | - |  | - | - | - |
| 75^th^ percentile | - | - | - |  | - | - | - |
| 90^th^ percentile | - | - | - |  | - | - | - |
| 95^th^ percentile | **0.451 (0.357; 0.570)** | **0.350 (0.253; 0.482)** | **0.554 (0.454; 0.675)** |  | **3.58 (2.96; 4.20)** | **3.62 (3.03; 4.20)** | **2.82 (2.34; 3.30)** |
| Maximum | - | - | - |  | - | - | - |
| Std deviation | - | 4.625 (1.332; 16.05) | - |  | -3.75 (-6.31; -1.19) | **-4.88 (-7.20; -2.56)** | **-4.14 (-5.79; -2.48)** |

Results are expressed as odds ratio and (95% confidence interval) for frailty and slope and (95% confidence interval) for handgrip. Statistical analysis using logistic regression for frailty and linear regression for handgrip. -, not retained. Results significant at P<0.001 are indicated in bold.

**Supplementary table S18:** Summary of the results for detection of frailty restricted to participants aged 65 years and older, CoLaus cohort, Lausanne, Switzerland, 2014-2017.

| **Name** | **Duration (s)** | **Speed metrics** | **AUC** | **LR** | **p-value** | **AIC** | **BIC** |
| --- | --- | --- | --- | --- | --- | --- | --- |
| Model A | Each duration | None | 0.709 |  |  | 869.3 | 899.1 |
| Model B | < 30 |  |  |  |  |  |  |
|  |  | Mode | 0.746 | 31.1 | <0.001 | 840.2 | 875.0 |
|  |  | Median | 0.749 | 37.5 | <0.001 | 833.8 | 868.6 |
|  |  | Mean | 0.756 | 45.0 | <0.001 | 826.3 | 861.1 |
|  |  | 75^th^ percentile | 0.755 | 43.6 | <0.001 | 827.7 | 862.4 |
|  |  | 90^th^ percentile | 0.761 | 50.5 | <0.001 | 820.8 | 855.5 |
|  |  | 95^th^ percentile | 0.760 | 49.8 | <0.001 | 821.5 | 856.3 |
|  |  | Maximum | 0.745 | 27.5 | <0.001 | 843.8 | 878.6 |
|  |  | Standard deviation | 0.741 | 30.1 | <0.001 | 841.2 | 876.0 |
|  | 30-120 |  |  |  |  |  |  |
|  |  | Mode | 0.743 | 28.1 | <0.001 | 843.2 | 878.0 |
|  |  | Median | 0.748 | 36.9 | <0.001 | 834.4 | 869.2 |
|  |  | Mean | 0.754 | 42.8 | <0.001 | 828.5 | 863.2 |
|  |  | 75^th^ percentile | 0.754 | 43.1 | <0.001 | 828.2 | 862.9 |
|  |  | 90^th^ percentile | 0.758 | 48.3 | <0.001 | 823.0 | 857.7 |
|  |  | 95^th^ percentile | 0.760 | 49.5 | <0.001 | 821.8 | 856.6 |
|  |  | Maximum | 0.740 | 26.1 | <0.001 | 845.2 | 879.9 |
|  |  | Standard deviation | 0.734 | 23.6 | <0.001 | 847.7 | 882.4 |
|  | > 120 |  |  |  |  |  |  |
|  |  | Mode | 0.730 | 15.3 | <0.001 | 856.0 | 890.7 |
|  |  | Median | 0.734 | 19.4 | <0.001 | 851.9 | 886.7 |
|  |  | Mean | 0.743 | 26.9 | <0.001 | 844.4 | 879.1 |
|  |  | 75^th^ percentile | 0.738 | 23.8 | <0.001 | 847.5 | 882.2 |
|  |  | 90^th^ percentile | 0.747 | 32.8 | <0.001 | 838.5 | 873.3 |
|  |  | 95^th^ percentile | 0.753 | 37.5 | <0.001 | 833.8 | 868.6 |
|  |  | Maximum | 0.726 | 12.1 | 0.001 | 859.2 | 893.9 |
|  |  | Standard deviation | 0.725 | 14.0 | <0.001 | 857.3 | 892.1 |

AUC area under the ROC curve; NaN the values which were not possible to be computed; AIC, Akaike's information criterion; BIC, Bayesian information criterion. Model A includes gender, age, BMI, and PA; model B consists of all variables from model A plus the variable of interest (the speed metric specified in each row). Models A and B were compared by likelihood ratio (LR) test.

**Supplementary Table S19:** Summary of the results for estimating handgrip strength restricted to participants aged 65 years and older, CoLaus cohort, Lausanne, Switzerland, 2014-2017.

| **Name** | **Duration (s)** | **Speed metrics** | **LR** | **P-value** | **AIC** | **BIC** |
| --- | --- | --- | --- | --- | --- | --- |
| Model A | Each duration | None | NaN | NaN | 7005.3 | 7035.1 |
| Model B | < 30 |  |  |  |  |  |
|  |  | Mode | 45.2 | <0.001 | 6962.2 | 6996.9 |
|  |  | Median | 72.5 | <0.001 | 6934.8 | 6969.6 |
|  |  | Mean | 92.8 | <0.001 | 6914.5 | 6949.3 |
|  |  | 75^th^ percentile | 75.7 | <0.001 | 6931.6 | 6966.4 |
|  |  | 90^th^ percentile | 96.9 | <0.001 | 6910.4 | 6945.2 |
|  |  | 95^th^ percentile | 101.3 | <0.001 | 6906.1 | 6940.8 |
|  |  | Maximum | 44.0 | <0.001 | 6963.4 | 6998.1 |
|  |  | Standard deviation | 42.8 | <0.001 | 6964.5 | 6999.3 |
|  | 30-120 |  |  |  |  |  |
|  |  | Mode | 53.4 | <0.001 | 7005.3 | 7035.1 |
|  |  | Median | 74.5 | <0.001 | 6954.0 | 6988.7 |
|  |  | Mean | 92.3 | <0.001 | 6932.8 | 6967.6 |
|  |  | 75^th^ percentile | 75.0 | <0.001 | 6915.1 | 6949.8 |
|  |  | 90^th^ percentile | 91.3 | <0.001 | 6932.4 | 6967.1 |
|  |  | 95^th^ percentile | 94.4 | <0.001 | 6916.1 | 6950.8 |
|  |  | Maximum | 32.3 | <0.001 | 6912.9 | 6947.7 |
|  |  | Standard deviation | 29.0 | <0.001 | 6975.0 | 7009.8 |
|  | > 120 |  |  |  |  |  |
|  |  | Mode | 30.4 | <0.001 | 6976.9 | 7011.6 |
|  |  | Median | 49.0 | <0.001 | 6958.4 | 6993.1 |
|  |  | Mean | 66.1 | <0.001 | 6941.2 | 6976.0 |
|  |  | 75^th^ percentile | 50.1 | <0.001 | 6957.2 | 6992.0 |
|  |  | 90^th^ percentile | 69.3 | <0.001 | 6938.0 | 6972.7 |
|  |  | 95^th^ percentile | 70.5 | <0.001 | 6936.9 | 6971.6 |
|  |  | Maximum | 27.2 | <0.001 | 6980.2 | 7014.9 |
|  |  | Standard deviation | 16.9 | <0.001 | 6990.5 | 7025.2 |

NaN the values which were not possible to be computed; AIC, Akaike's information criterion; BIC, Bayesian information criterion. Model A includes gender, age categories, BMI categories, and PA; model B consists of all variables from model A plus the variable of interest (the speed metric specified in each row). Models A and B were compared by likelihood ratio (LR) test.

**Supplementary Table S20:** Summary of the re-analysis of the stepwise regression restricted to participants aged 65 years and older, CoLaus cohort, Lausanne, Switzerland, 2014-2017.

| **Speed metrics** | ***P*-values for frailty** | | | ***P*-values for Handgrip strength** | | |
| --- | --- | --- | --- | --- | --- | --- |
|  | **< 30 s** | **30-120 s** | **> 120 s** | **< 30 s** | **30-120 s** | **> 120 s** |
| Mode | - | - | - | - | - | - |
| Median | - | - | - | - | - | - |
| Mean | - | - | - | - | - | - |
| 75^th^ percentile | - | - | - | - | - | - |
| 90^th^ percentile | **<0.001** | - | - | - | - | - |
| 95^th^ percentile | **-** | **<0.001** | **<0.001** | **<0.001** | **<0.001** | **<0.001** |
| Maximum | 0.040 | - | - | - | - | - |
| Standard deviation | - | - | - | - | - | 0.009 |

s, seconds; '-' the variable was not retained in the stepwise approach. The table reports the *P*-values obtained by using each speed metric within each duration. *P*-values <0.001 are indicated in bold.

**Supplementary table S21**: full results to predict frailty, baseline model and models including the different metrics for bout periods <30 seconds, CoLaus cohort, Lausanne, Switzerland, 2014-2017. Analysis restricted to participants aged 65 years and older.

|  | **Baseline** | **+ mode** | **+ median** | **+ mean** | **+ 75^th^ percentile** | **+ 90^th^ percentile** | **+ 95^th^ percentile** | **+ maximum** | **+ std deviation** |
| --- | --- | --- | --- | --- | --- | --- | --- | --- | --- |
| Man vs. woman | 0.55 (0.38-0.78) | 0.80 (0.55-1.18) | 1.01 (0.67-1.53) | 1.24 (0.80-1.91) | 1.14 (0.75-1.74) | 1.47 (0.93-2.33) | 1.58 (0.99-2.53) | 0.88 (0.58-1.32) | 0.99 (0.65-1.51) |
| Age categories |  |  |  |  |  |  |  |  |  |
| [65-75[ | 1 (ref.) | 1 (ref.) | 1 (ref.) | 1 (ref.) | 1 (ref.) | 1 (ref.) | 1 (ref.) | 1 (ref.) | 1 (ref.) |
| [75+ | **2.88 (2.04-4.07)** | **2.56 (1.80-3.64)** | **2.62 (1.84-3.73)** | **2.58 (1.81-3.68)** | **2.71 (1.90-3.86)** | **2.63 (1.84-3.75)** | **2.54 (1.78-3.63)** | **2.77 (1.96-3.94)** | **2.8 (1.97-3.97)** |
| BMI categories |  |  |  |  |  |  |  |  |  |
| Normal | 1 (ref.) | 1 (ref.) | 1 (ref.) | 1 (ref.) | 1 (ref.) | 1 (ref.) | 1 (ref.) | 1 (ref.) | 1 (ref.) |
| Overweight | 1.33 (0.87-2.03) | 1.26 (0.82-1.93) | 1.21 (0.79-1.86) | 1.18 (0.76-1.81) | 1.17 (0.76-1.81) | 1.13 (0.73-1.75) | 1.13 (0.73-1.75) | 1.17 (0.76-1.80) | 1.15 (0.74-1.77) |
| Obese | **2.78 (1.76-4.38)** | **2.78 (1.76-4.42)** | **2.68 (1.69-4.27)** | **2.51 (1.57-4.00)** | **2.54 (1.59-4.05)** | 2.26 (1.41-3.61) | 2.20 (1.38-3.53) | **2.38 (1.50-3.79)** | 2.22 (1.39-3.55) |
| MVPA § | 0.41 (0.24-0.72) | 0.50 (0.28-0.88) | 0.51 (0.29-0.91) | 0.49 (0.28-0.86) | 0.51 (0.29-0.89) | 0.45 (0.26-0.80) | 0.42 (0.24-0.75) | 0.38 (0.22-0.67) | 0.41 (0.23-0.72) |
| Marker ‡ | **0.32 (0.19-0.55)** | **0.50 (0.39-0.64)** | **0.41 (0.30-0.55)** | **0.36 (0.26-0.49)** | **0.43 (0.34-0.56)** | **0.43 (0.34-0.55)** | **0.45 (0.36-0.57)** | **0.79 (0.71-0.87)** | **0.06 (0.02-0.18)** |
| AIC | 869.3 | 840.2 | 833.8 | 826.3 | 827.7 | 820.8 | 821.5 | 843.8 | 841.2 |
| BIC | 899.1 | 875.0 | 868.6 | 861.1 | 862.4 | 855.5 | 856.3 | 878.6 | 876.0 |
| AUC | 0.709 | 0.746 | 0.749 | 0.756 | 0.755 | 0.761 | 0.760 | 0.745 | 0.741 |
| LR-test † |  | 31.1 | 37.5 | 45.0 | 43.6 | 50.5 | 49.8 | 27.5 | 30.1 |
| p-value |  | <0.001 | <0.001 | <0.001 | <0.001 | <0.001 | <0.001 | <0.001 | <0.001 |

§ ≥150 vs. < 150 min/week; ‡ per 0.1 unit increase; †, compared to the baseline model. AIC, Akaike information criterion; AUC, area under the ROC; BIC, Bayesian information criterion. Results are expressed as odds-ratio and (95% confidence interval). Statistical analysis using logistic regression; significant results at P<0.001 are indicated in bold.

**Supplementary table S22**: full results to predict frailty, baseline model and models including the different metrics for bout periods between 30 and 120 seconds, CoLaus cohort, Lausanne, Switzerland, 2014-2017. Analysis restricted to participants aged 65 years and older.

|  | **Baseline** | **+ mode** | **+ median** | **+ mean** | **+ 75^th^ percentile** | **+ 90^th^ percentile** | **+ 95^th^ percentile** | **+ maximum** | **+ std deviation** |
| --- | --- | --- | --- | --- | --- | --- | --- | --- | --- |
| Man vs. woman | 0.55 (0.38-0.78) | 0.78 (0.53-1.14) | 1.00 (0.66-1.50) | 1.20 (0.78-1.85) | 1.15 (0.75-1.75) | 1.46 (0.92-2.30) | 1.58 (0.99-2.53) | 0.82 (0.55-1.23) | 0.92 (0.61-1.40) |
| Age categories |  |  |  |  |  |  |  |  |  |
| [65-75[ | 1 (ref.) | 1 (ref.) | 1 (ref.) | 1 (ref.) | 1 (ref.) | 1 (ref.) | 1 (ref.) | 1 (ref.) | 1 (ref.) |
| [75+ | **2.88 (2.04-4.07)** | **2.63 (1.85-3.74)** | **2.63 (1.85-3.75)** | **2.61 (1.83-3.72)** | **2.73 (1.92-3.88)** | **2.65 (1.86-3.77)** | **2.54 (1.78-3.63)** | **2.78 (1.96-3.94)** | **2.78 (1.96-3.94)** |
| BMI categories |  |  |  |  |  |  |  |  |  |
| Normal | 1 (ref.) | 1 (ref.) | 1 (ref.) | 1 (ref.) | 1 (ref.) | 1 (ref.) | 1 (ref.) | 1 (ref.) | 1 (ref.) |
| Overweight | 1.33 (0.87-2.03) | 1.25 (0.82-1.92) | 1.19 (0.78-1.84) | 1.16 (0.75-1.79) | 1.16 (0.75-1.80) | 1.12 (0.72-1.73) | 1.10 (0.71-1.70) | 1.19 (0.78-1.83) | 1.18 (0.77-1.82) |
| Obese | **2.78 (1.76-4.38)** | **2.83 (1.78-4.48)** | **2.63 (1.66-4.19)** | **2.46 (1.54-3.92)** | **2.50 (1.57-3.99)** | 2.22 (1.39-3.56) | 2.14 (1.34-3.43) | **2.42 (1.52-3.84)** | **2.30 (1.44-3.67)** |
| MVPA § | 0.41 (0.24-0.72) | 0.49 (0.28-0.87) | 0.51 (0.29-0.90) | 0.49 (0.28-0.86) | 0.51 (0.29-0.90) | 0.46 (0.26-0.80) | 0.42 (0.24-0.75) | 0.38 (0.22-0.67) | 0.41 (0.23-0.71) |
| Marker ‡ | **0.32 (0.19-0.55)** | **0.52 (0.41-0.67)** | **0.42 (0.32-0.56)** | **0.38 (0.28-0.51)** | **0.44 (0.34-0.57)** | **0.45 (0.35-0.57)** | **0.46 (0.37-0.58)** | **0.79 (0.71-0.87)** | **0.10 (0.04-0.26)** |
| AIC | 869.3 | 843.2 | 834.4 | 828.5 | 828.2 | 823.0 | 821.8 | 845.2 | 847.7 |
| BIC | 899.1 | 878.0 | 869.2 | 863.2 | 862.9 | 857.7 | 856.6 | 879.9 | 882.4 |
| AUC | 0.709 | 0.743 | 0.748 | 0.754 | 0.754 | 0.758 | 0.760 | 0.740 | 0.734 |
| LR-test † |  | 28.1 | 36.9 | 42.8 | 43.1 | 48.3 | 49.5 | 26.1 | 23.6 |
| p-value |  | <0.001 | <0.001 | <0.001 | <0.001 | <0.001 | <0.001 | <0.001 | <0.001 |

§ ≥150 vs. < 150 min/week; ‡ per 0.1 unit increase; †, compared to the baseline model. AIC, Akaike information criterion; AUC, area under the ROC; BIC, Bayesian information criterion. Results are expressed as odds-ratio and (95% confidence interval). Statistical analysis using logistic regression; significant results at P<0.001 are indicated in bold.

**Supplementary table S23**: full results to predict frailty, baseline model and models including the different metrics for bout periods above 120 seconds, CoLaus cohort, Lausanne, Switzerland, 2014-2017. Analysis restricted to participants aged 65 years and older.

|  | **Baseline** | **+ mode** | **+ median** | **+ mean** | **+ 75^th^ percentile** | **+ 90^th^ percentile** | **+ 95^th^ percentile** | **+ maximum** | **+ std deviation** |
| --- | --- | --- | --- | --- | --- | --- | --- | --- | --- |
| Man vs. woman | 0.55 (0.38-0.78) | 0.72 (0.49-1.05) | 0.83 (0.55-1.24) | 0.97 (0.64-1.48) | 0.88 (0.59-1.33) | 1.10 (0.71-1.70) | 1.21 (0.78-1.90) | 0.73 (0.49-1.09) | 0.76 (0.51-1.12) |
| Age categories |  |  |  |  |  |  |  |  |  |
| [65-75[ | 1 (ref.) | 1 (ref.) | 1 (ref.) | 1 (ref.) | 1 (ref.) | 1 (ref.) | 1 (ref.) | 1 (ref.) | 1 (ref.) |
| [75+ | **2.88 (2.04-4.07)** | **2.77 (1.96-3.93)** | **2.73 (1.93-3.87)** | **2.65 (1.87-3.77)** | **2.71 (1.91-3.84)** | **2.59 (1.82-3.68)** | **2.51 (1.76-3.57)** | **2.68 (1.89-3.80)** | **2.69 (1.90-3.82)** |
| BMI categories |  |  |  |  |  |  |  |  |  |
| Normal | 1 (ref.) | 1 (ref.) | 1 (ref.) | 1 (ref.) | 1 (ref.) | 1 (ref.) | 1 (ref.) | 1 (ref.) | 1 (ref.) |
| Overweight | 1.33 (0.87-2.03) | 1.23 (0.80-1.89) | 1.21 (0.79-1.85) | 1.17 (0.76-1.80) | 1.18 (0.77-1.81) | 1.15 (0.74-1.77) | 1.14 (0.74-1.75) | 1.26 (0.83-1.93) | 1.22 (0.79-1.87) |
| Obese | **2.78 (1.76-4.38)** | **2.65 (1.68-4.18)** | **2.62 (1.66-4.15)** | **2.52 (1.59-3.99)** | **2.54 (1.60-4.03)** | **2.32 (1.46-3.69)** | **2.30 (1.44-3.66)** | **2.62 (1.65-4.14)** | **2.46 (1.55-3.90)** |
| MVPA § | 0.41 (0.24-0.72) | 0.48 (0.27-0.84) | 0.47 (0.27-0.83) | 0.46 (0.26-0.81) | 0.46 (0.26-0.81) | 0.43 (0.24-0.75) | 0.42 (0.24-0.74) | 0.42 (0.24-0.73) | 0.40 (0.23-0.71) |
| Marker ‡ | **0.32 (0.19-0.55)** | **0.66 (0.53-0.81)** | **0.58 (0.45-0.74)** | **0.51 (0.39-0.66)** | **0.60 (0.49-0.74)** | **0.57 (0.47-0.70)** | **0.57 (0.47-0.69)** | **0.83 (0.75-0.93)** | **0.26 (0.13-0.53)** |
| AIC | 869.3 | 856.0 | 851.9 | 844.4 | 847.5 | 838.5 | 833.8 | 859.2 | 857.3 |
| BIC | 899.1 | 890.7 | 886.7 | 879.1 | 882.2 | 873.3 | 868.6 | 893.9 | 892.1 |
| AUC | 0.709 | 0.730 | 0.734 | 0.743 | 0.738 | 0.747 | 0.753 | 0.726 | 0.725 |
| LR-test † |  | 15.3 | 19.4 | 26.9 | 23.8 | 32.8 | 37.5 | 12.1 | 14.0 |
| p-value |  | <0.001 | <0.001 | <0.001 | <0.001 | <0.001 | <0.001 | 0.001 | <0.001 |

§ ≥150 vs. < 150 min/week; ‡ per 0.1 unit increase; †, compared to the baseline model. AIC, Akaike information criterion; AUC, area under the ROC; BIC, Bayesian information criterion. Results are expressed as odds-ratio and (95% confidence interval). Statistical analysis using logistic regression; significant results at P<0.001 are indicated in bold.

**Supplementary table S24**: full results to predict handgrip, baseline model and models including the different metrics for bout periods <30 seconds, CoLaus cohort, Lausanne, Switzerland, 2014-2017. Analysis restricted to participants aged 65 years and older.

|  | **Baseline** | **+ mode** | **+ median** | **+ mean** | **+ 75^th^ percentile** | **+ 90^th^ percentile** | **+ 95^th^ percentile** | **+ maximum** | **+ std deviation** |
| --- | --- | --- | --- | --- | --- | --- | --- | --- | --- |
| Man vs. woman | **16.3 (15.5; 17.1)** | **15.3 (14.4; 16.2)** | **14.4 (13.5; 15.3)** | **13.7 (12.8; 14.7)** | **14.2 (13.2; 15.1)** | **13.3 (12.3; 14.3)** | **13.0 (12.0; 14.0)** | **15.2 (14.3; 16.0)** | **14.8 (13.8; 15.7)** |
| Age categories |  |  |  |  |  |  |  |  |  |
| [65-75[ | 1 (ref.) | 1 (ref.) | 1 (ref.) | 1 (ref.) | 1 (ref.) | 1 (ref.) | 1 (ref.) | 1 (ref.) | 1 (ref.) |
| [75+ | **-3.6 (-4.5; -2.7)** | **-3.2 (-4.0; -2.3)** | **-3.1 (-4.0; -2.3)** | **-3.0 (-3.9; -2.2)** | **-3.2 (-4.1; -2.4)** | **-3.1 (-3.9; -2.3)** | **-3.0 (-3.8; -2.1)** | **-3.4 (-4.3; -2.6)** | **-3.4 (-4.3; -2.6)** |
| BMI categories |  |  |  |  |  |  |  |  |  |
| Normal | 1 (ref.) | 1 (ref.) | 1 (ref.) | 1 (ref.) | 1 (ref.) | 1 (ref.) | 1 (ref.) | 1 (ref.) | 1 (ref.) |
| Overweight | 0.8 (-0.1; 1.7) | 0.9 (0; 1.8) | 1.1 (0.2; 2.0) | 1.2 (0.3; 2.1) | 1.2 (0.3; 2.1) | 1.4 (0.5; 2.2) | 1.4 (0.5; 2.3) | 1.1 (0.2; 2.0) | 1.3 (0.3; 2.2) |
| Obese | 0.8 (-0.4; 1.9) | 0.9 (-0.3; 2.0) | 1.0 (-0.1; 2.1) | 1.3 (0.2; 2.4) | 1.2 (0.1; 2.3) | 1.6 (0.5; 2.7) | 1.7 (0.6; 2.8) | 1.2 (0.1; 2.3) | 1.5 (0.3; 2.6) |
| MVPA § | 1.0 (0; 2.0) | 0.5 (-0.5; 1.5) | 0.4 (-0.6; 1.3) | 0.5 (-0.5; 1.5) | 0.4 (-0.5; 1.4) | 0.7 (-0.2; 1.7) | 0.9 (-0.1; 1.8) | **1.3 (0.3; 2.2)** | 1.0 (0; 2.0) |
| Marker ‡ |  | **1.8 (1.3; 2.3)** | **2.7 (2.1; 3.3)** | **3.2 (2.5; 3.8)** | **2.4 (1.9; 2.9)** | **2.5 (2.0; 3.0)** | **2.4 (2.0; 2.9)** | **0.6 (0.4; 0.8)** | **7 (4.9; 9.1)** |
| AIC | 7005.3 | 6962.2 | 6934.8 | 6914.5 | 6931.6 | 6910.4 | 6906.1 | 6963.4 | 6964.5 |
| BIC | 7035.1 | 6996.9 | 6969.6 | 6949.3 | 6966.4 | 6945.2 | 6940.8 | 6998.1 | 6999.3 |
| Adjusted R^2^ | 0.608 | 0.625 | 0.634 | 0.641 | 0.635 | 0.642 | 0.644 | 0.624 | 0.624 |
| LR-test † |  | 45.2 | 72.5 | 92.8 | 75.7 | 96.9 | 101.3 | 44 | 42.8 |
| p-value |  | <0.001 | <0.001 | <0.001 | <0.001 | <0.001 | <0.001 | <0.001 | <0.001 |

§ ≥150 vs. < 150 min/week; ‡ per 0.1 unit increase; †, compared to the baseline model. AIC, Akaike information criterion; BIC, Bayesian information criterion. Results are expressed as slope and (95% confidence interval). Statistical analysis using linear regression; significant results at P<0.001 are indicated in bold.

**Supplementary table S25**: full results to predict handgrip, baseline model and models including the different metrics for bout periods between 30 and 120 seconds, CoLaus cohort, Lausanne, Switzerland, 2014-2017. Analysis restricted to participants aged 65 years and older.

|  | **Baseline** | **+ mode** | **+ median** | **+ mean** | **+ 75^th^ percentile** | **+ 90^th^ percentile** | **+ 95^th^ percentile** | **+ maximum** | **+ std deviation** |
| --- | --- | --- | --- | --- | --- | --- | --- | --- | --- |
| Man vs. woman | **16.3 (15.5; 17.1)** | **15.2 (14.4; 16.1)** | **14.4 (13.5; 15.3)** | **13.8 (12.8; 14.7)** | **14.1 (13.2; 15.1)** | **13.4 (12.4; 14.4)** | **13.1 (12.1; 14.1)** | **15.4 (14.6; 16.3)** | **15.1 (14.1; 16.0)** |
| Age categories |  |  |  |  |  |  |  |  |  |
| [65-75[ | 1 (ref.) | 1 (ref.) | 1 (ref.) | 1 (ref.) | 1 (ref.) | 1 (ref.) | 1 (ref.) | 1 (ref.) | 1 (ref.) |
| [75+ | **-3.6 (-4.5; -2.7)** | **-3.2 (-4.0; -2.3)** | **-3.1 (-4.0; -2.3)** | **-3.1 (-3.9; -2.2)** | **-3.2 (-4.1; -2.4)** | **-3.1 (-4.0; -2.3)** | **-3.0 (-3.8; -2.1)** | **-3.5 (-4.3; -2.6)** | **-3.4 (-4.3; -2.6)** |
| BMI categories |  |  |  |  |  |  |  |  |  |
| Normal | 1 (ref.) | 1 (ref.) | 1 (ref.) | 1 (ref.) | 1 (ref.) | 1 (ref.) | 1 (ref.) | 1 (ref.) | 1 (ref.) |
| Overweight | 0.8 (-0.1; 1.7) | 1.0 (0; 1.9) | 1.1 (0.2; 2.0) | 1.2 (0.3; 2.1) | 1.2 (0.3; 2.1) | 1.4 (0.5; 2.3) | 1.4 (0.5; 2.3) | 1.0 (0.1; 1.9) | 1.2 (0.3; 2.1) |
| Obese | 0.8 (-0.4; 1.9) | 0.8 (-0.3; 1.9) | 1.1 (0; 2.2) | 1.3 (0.2; 2.4) | 1.2 (0.1; 2.3) | 1.7 (0.5; 2.8) | 1.8 (0.7; 2.9) | 1.1 (0; 2.2) | 1.3 (0.2; 2.5) |
| MVPA § | 1 (0; 2.0) | 0.5 (-0.5; 1.4) | 0.4 (-0.6; 1.3) | 0.5 (-0.5; 1.4) | 0.4 (-0.5; 1.4) | 0.7 (-0.2; 1.7) | 0.9 (0; 1.9) | **1.2 (0.2; 2.2)** | **1 (0; 2.0)** |
| Marker ‡ |  | **1.9 (1.4; 2.4)** | **2.7 (2.1; 3.3)** | **3.1 (2.5; 3.7)** | **2.3 (1.8; 2.9)** | **2.4 (1.9; 2.9)** | **2.3 (1.9; 2.8)** | **0.5 (0.3; 0.7)** | **5.4 (3.4; 7.4)** |
| AIC | 7005.3 | 6954.0 | 6932.8 | 6915.1 | 6932.4 | 6916.1 | 6912.9 | 6975.0 | 6978.4 |
| BIC | 7035.1 | 6988.7 | 6967.6 | 6949.8 | 6967.1 | 6950.8 | 6947.7 | 7009.8 | 7013.1 |
| Adjusted R^2^ | 0.608 | 0.627 | 0.635 | 0.641 | 0.635 | 0.641 | 0.642 | 0.620 | 0.619 |
| LR-test † |  | 53.4 | 74.5 | 92.3 | 75.0 | 91.3 | 94.4 | 32.3 | 29.0 |
| p-value |  | <0.001 | <0.001 | <0.001 | <0.001 | <0.001 | <0.001 | <0.001 | <0.001 |

§ ≥150 vs. < 150 min/week; ‡ per 0.1 unit increase; †, compared to the baseline model. AIC, Akaike information criterion; BIC, Bayesian information criterion. Results are expressed as slope and (95% confidence interval). Statistical analysis using linear regression; significant results are indicated in bold.

**Supplementary table S26**: full results to predict handgrip, baseline model and models including the different metrics for bout periods above 120 seconds, CoLaus cohort, Lausanne, Switzerland, 2014-2017. Analysis restricted to participants aged 65 years and older.

|  | **Baseline** | **+ mode** | **+ median** | **+ mean** | **+ 75^th^ percentile** | **+ 90^th^ percentile** | **+ 95^th^ percentile** | **+ maximum** | **+ std deviation** |
| --- | --- | --- | --- | --- | --- | --- | --- | --- | --- |
| Man vs. woman | **16.3 (15.5; 17.1)** | **15.5 (14.6; 16.3)** | **14.9 (14.0; 15.8)** | **14.3 (13.4; 15.3)** | **14.8 (13.9; 15.7)** | **14.1 (13.1; 15.0)** | **14.0 (13.0; 14.9)** | **15.4 (14.5; 16.2)** | **15.5 (14.6; 16.4)** |
| Age categories |  |  |  |  |  |  |  |  |  |
| [65-75[ | 1 (ref.) | 1 (ref.) | 1 (ref.) | 1 (ref.) | 1 (ref.) | 1 (ref.) | 1 (ref.) | 1 (ref.) | 1 (ref.) |
| [75+ | **-3.6 (-4.5; -2.7)** | **-3.4 (-4.3; -2.6)** | **-3.3 (-4.2; -2.5)** | **-3.2 (-4.0; -2.3)** | **-3.3 (-4.1; -2.4)** | **-3.1 (-3.9; -2.2)** | **-3.0 (-3.9; -2.2)** | **-3.3 (-4.2; -2.5)** | **-3.4 (-4.3; -2.5)** |
| BMI categories |  |  |  |  |  |  |  |  |  |
| Normal | 1 (ref.) | 1 (ref.) | 1 (ref.) | 1 (ref.) | 1 (ref.) | 1 (ref.) | 1 (ref.) | 1 (ref.) | 1 (ref.) |
| Overweight | 0.8 (-0.1; 1.7) | 1.0 (0.1; 1.9) | 1.1 (0.2; 2.0) | 1.2 (0.3; 2.1) | 1.2 (0.3; 2.1) | 1.3 (0.4; 2.2) | 1.3 (0.4; 2.2) | 1.0 (0.1; 1.9) | 1.1 (0.1; 2.0) |
| Obese | 0.8 (-0.4; 1.9) | 1.0 (-0.1; 2.1) | 1.0 (-0.1; 2.2) | 1.2 (0.1; 2.3) | 1.1 (0; 2.2) | 1.4 (0.3; 2.6) | 1.5 (0.3; 2.6) | 1.0 (-0.1; 2.2) | 1.1 (0; 2.2) |
| MVPA § | 1 (0; 2.0) | 0.5 (-0.5; 1.5) | 0.5 (-0.5; 1.5) | 0.6 (-0.4; 1.5) | 0.6 (-0.3; 1.6) | 0.8 (-0.1; 1.8) | 0.9 (-0.1; 1.9) | 1.0 (0; 2.0) | **1.1 (0.1; 2.0)** |
| Marker ‡ |  | **1.3 (0.8; 1.8)** | **1.9 (1.4; 2.5)** | **2.3 (1.8; 2.9)** | **1.6 (1.2; 2.1)** | **1.8 (1.4; 2.2)** | **1.7 (1.3; 2.1)** | **0.6 (0.4; 0.8)** | **3.3 (1.7; 4.9)** |
| AIC | 7005.3 | 6976.9 | 6958.4 | 6941.2 | 6957.2 | 6938.0 | 6936.9 | 6980.2 | 6990.5 |
| BIC | 7035.1 | 7011.6 | 6993.1 | 6976.0 | 6992.0 | 6972.7 | 6971.6 | 7014.9 | 7025.2 |
| Adjusted R^2^ | 0.608 | 0.620 | 0.626 | 0.632 | 0.626 | 0.633 | 0.633 | 0.618 | 0.614 |
| LR-test † |  | 30.4 | 49.0 | 66.1 | 50.1 | 69.3 | 70.5 | 27.2 | 16.9 |
| p-value |  | <0.001 | <0.001 | <0.001 | <0.001 | <0.001 | <0.001 | <0.001 | <0.001 |

§ ≥150 vs. < 150 min/week; ‡ per 0.1 unit increase; †, compared to the baseline model. AIC, Akaike information criterion; BIC, Bayesian information criterion. Results are expressed as slope and (95% confidence interval). Statistical analysis using linear regression; significant results at P<0.001 are indicated in bold.

**Supplementary table S27**: stepwise analysis to predict frailty or handgrip, according to bout period, CoLaus cohort, Lausanne, Switzerland, 2014-2017. Analysis restricted to participants aged 65 years and older.

|  |  | **Frailty** |  |  |  | **Handgrip** |  |
| --- | --- | --- | --- | --- | --- | --- | --- |
|  | **<30 seconds** | **30-120 seconds** | **>120 seconds** |  | **<30 seconds** | **30-120 seconds** | **>120 seconds** |
| Mode | - | - | - |  | - | - | - |
| Median | - | - | - |  | - | - | - |
| Mean | - | - | - |  | - | - | - |
| 75^th^ percentile | - | - | - |  | - | - | - |
| 90^th^ percentile | **0.490 (0.374; 0.643)** |  |  |  | - | - | - |
| 95^th^ percentile | - | **0.459 (0.365; 0.576)** | **0.567 (0.469; 0.687)** |  | **2.45 (1.98; 2.92)** | **2.31 (1.85; 2.77)** | **2.20 (1.65; 2.75)** |
| Maximum | 0.899 (0.813; 0.995) | - | - |  | - | - | - |
| Std deviation | - | - | - |  | - | - | -2.96 (-5.16; -0.76) |

Results are expressed as odds ratio and (95% confidence interval) for frailty and slope and (95% confidence interval) for handgrip. Statistical analysis using logistic regression for frailty and linear regression for handgrip. -, not retained. Results significant at P<0.001 are indicated in bold.
